# Functional Diversification Analysis of Soybean Malectin/Malectin-Like Domain-Containing Receptor-Like Kinases in Immunity by Transient Expression Assays

**DOI:** 10.3389/fpls.2022.938876

**Published:** 2022-06-23

**Authors:** Qian Zhang, Shuxian Chen, Yazhou Bao, Dongmei Wang, Weijie Wang, Rubin Chen, Yixin Li, Guangyuan Xu, Xianzhong Feng, Xiangxiu Liang, Daolong Dou

**Affiliations:** ^1^MOA Key Lab of Pest Monitoring and Green Management, Department of Plant Pathology, College of Plant Protection, China Agricultural University, Beijing, China; ^2^Key Laboratory of Soybean Molecular Design Breeding, Northeast Institute of Geography and Agroecology, Innovative Academy of Seed Design, Chinese Academy of Sciences, Changchun, China; ^3^College of Life Sciences, South China Agricultural University, Guangzhou, China; ^4^College of Plant Protection, Nanjing Agricultural University, Nanjing, China

**Keywords:** soybean, malectin/malectin-like domain-containing receptor-like kinases, immune responses, PTI, ETI

## Abstract

Plants have responded to microbial pathogens by evolving a two-tiered immune system, involving pathogen-associated molecular pattern (PAMP)-triggered immunity (PTI) and effector-triggered immunity (ETI). Malectin/malectin-like domain-containing receptor-like kinases (MRLKs) have been reported to participate in many biological functions in plant including immunity and resistance. However, little is known regarding the role of MRLKs in soybean immunity. This is a crucial question to address because soybean is an important source of oil and plant proteins, and its production is threatened by various pathogens. Here, we systematically identified 72 *Glycine max* MRLKs (GmMRLKs) and demonstrated that many of them are transcriptionally induced or suppressed in response to infection with microbial pathogens. Next, we successfully cloned 60 *GmMRLKs* and subsequently characterized their roles in plant immunity by transiently expressing them in *Nicotiana benthamiana*, a model plant widely used to study host-pathogen interactions. Specifically, we examined the effect of GmMRLKs on PTI responses and noticed that a number of GmMRLKs negatively regulated the reactive oxygen species burst induced by flg22 and chitin, and cell death triggered by XEG1 and INF1. We also analyzed the microbial effectors AvrB- and XopQ-induced hypersensitivity response and identified several GmMRLKs that suppressed ETI activation. We further showed that GmMRLKs regulate immunity probably by coupling to the immune receptor complexes. Furthermore, transient expression of several selected GmMRLKs in soybean hairy roots conferred reduced resistance to soybean pathogen *Phytophthora sojae.* In summary, we revealed the common and specific roles of GmMRLKs in soybean immunity and identified a number of *GmMRLKs* as candidate susceptible genes that may be useful for improving soybean resistance.

## Introduction

Soybean (*Glycine max*) is a major source of plant oil, but its production is threatened by various pathogens, including Phytophthora root rot, bacterial blight, and soybean rust. Thus, it is important to advance our understanding of the soybean immune system and to improve soybean resistance. To defend against microbial pathogens, plants have evolved a complex innate immune system to protect themselves from infections. Plant cells rely on plasma membrane-localized pattern-recognition receptors (PRRs) and intracellular nucleotide-binding, leucine-rich repeat receptors (NLRs) that perceive microbial invasion and activate plant immunity. PRRs mainly consist of receptor-like kinases (RLKs) and receptor-like proteins (RLPs) that recognize pathogen/microbe-associated patterns (PAMPs/MAMPs) to initiate PAMP/MAMP-triggered immunity (PTI/MTI) (Tang et al., 2017; DeFalco and Zipfel, 2021). For example, Arabidopsis FLS2 recognizes flagellin 22 (flg22) in the presence of the co-receptor BAK1 (Chinchilla et al., 2007; Sun et al., 2013). Fungal cell wall-derived chitin is recognized by the Arabidopsis RLK proteins, CERK1 and LYK4/5 (Miya et al., 2007; Cao et al., 2014). *Nicotiana benthamiana (N. benthamiana)* RLP protein RXEG1 recognizes *Phytophthora sojae* (*P. sojae*) XEG1 in the presence of the co-receptors NbBAK1 and NbSOBIR1 (Ma et al., 2017; Wang et al., 2018). Additionally, *P. infestans*-derived INF1 is recognized by the RLP protein ELR in potato in a BAK1- and SOBIR1- dependent manner (Du et al., 2015). PRRs transduce immune signals through many regulatory components, such as BIK1 and related PBS1-like proteins (Lu et al., 2010; Zhang et al., 2010), heterotrimeric G proteins (Liang et al., 2016, 2018), and NADPH oxidase RbohD (Kadota et al., 2014; Li et al., 2014), which form a complex with PRRs. PAMPs induce a series of downstream immune responses, including the transient production of reactive oxygen species (ROS) and calcium influx, callose deposition, activation of mitogen-activated protein kinase (MAPK) and calcium-dependent protein kinase (CDPK), and transcriptional programming to achieve PTI activation (DeFalco and Zipfel; Jones and Dangl, 2006). Certain PAMPs can even trigger cell death in plants, such as XEG1 and INF1 induce cell death in *N. benthamiana* plants (Kamoun et al., 1998; Ma et al., 2017).

Successful pathogens can evade plant PTI by secreting effectors into host cells, which suppress plant immunity and consequently facilitate pathogen infection. For example, the *Pseudmonas syringae* (*P. syringae*) effector HopAI1 inactivates MAPKs through its phosphothreonine lyase activity (Zhang et al., 2007). Another *P. syringae* effector AvrB leads to the phosphorylation of RIN4 to positively regulate the H^+^-ATPase AHA1 (Chung et al., 2011; Liu J. et al., 2011), which enhances the jasmonic acid signaling to regulate stomatal opening (Zhou et al., 2015). Further, NIS1, a conserved effector in filamentous fungi, targets and inhibits the kinase activities of BAK1 and BIK1 to suppress host immune activation (Irieda et al., 2019). The Oomycete RXLR effector RXLR25 targets RLCK subfamily VII (RLCK-VII) proteins and inhibits the phosphorylation of RLCK-VII proteins (Liang et al., 2021).

To overcome the dampening of plant immunity by microbial effectors, plant intracellular NLRs recognize the microbial effectors and activate a second round of plant responses to further amplify and strengthen plant immunity (Jones et al., 2016). NLRs mainly consist of toll/IL-1 receptor-NLRs (TNLs) and coiled-coil NLRs (CNLs), and plants possess a limited number of NLRs that recognize numerous effectors (Jones et al., 2016; Zhou et al., 2020). Plant NLR recognizes effectors through direct interactions and multiple indirect strategies (Jones et al., 2016). Thus, most effectors are recognized by NLRs through indirect interactions. Additionally, a number of NLRs recognize effectors by monitoring host proteins that are targeted and modified by pathogen effectors. These host proteins can either be virulence targets (guard model) or mimic the virulence targets (decoy model) of effectors (van der Hoorn and Kamoun, 2008; Zhou and Chai, 2008). For example, *P. syringae* effector AvrB targets and leads to the phosphorylation of RIN4 in the presence of host RIPK kinase, and the CNL protein RPM1 monitors the changes in RIN4 and is activated to triggered the hypersensitive response (HR) in Arabidopsis and *N. benthamiana* (Grant et al., 1995; Mackey et al., 2002; Chung et al., 2011; Liu T. et al., 2011). Similarly, AvrB is recognized by the CNL protein, RPG1B, in the presence of GmRIN4a or GmRIN4b (Ashfield et al., 2004; Selote and Kachroo, 2010). The *Xanthomonas* effector AvrAC targets and uridylates PBL2, which is recruited by the RKS1-ZAR1 complex and forms a resistosome (Wang et al., 2015, 2019a,b), which serves as a calcium channel that triggers ETI and HR (Bi et al., 2021). Another *Xanthomonas* effector, XopQ, is directly recognized by the TNL protein ROQ1 by forming a tetrameric resistosome (Schultink et al., 2017; Martin et al., 2020). Recent studies have shown that PTI is essential for ETI activation, which in turn activates immunity by augmenting PTI (Ngou et al., 2021; Yuan et al., 2021). Both NLR- and PRR-mediated immunity share many regulatory machineries, including BIK1, RbohD, and MAPK cascades, which are important for activating both layers of immunity (Ngou et al., 2021; Pruitt et al., 2021; Tian et al., 2021; Yuan et al., 2021).

Malectin/malectin-like domain containing receptor-like kinases (MRLKs) are RLKs with extracellular malectin or malectin-like motifs (Dievart et al., 2020). MRLKs play pivotal roles in plant immunity and can be divided into three groups: malectin-like kinases (also known as CrRLK1L kinases), malectin-like leucine-rich repeat (LRR) kinases, and LRR malectin kinases (Dievart et al., 2020). Most CrRLK1L kinases are involved in the recognition of a family of (rapid alkalinization factor) RALF peptides (Franck et al., 2018), and have been extensively studied in the last decade for their multiple roles in plant growth, reproduction, and responses to abiotic and biotic stresses (Franck et al., 2018). FERONIA (FER) and IOS1 are the most well-studied MRLKs that modulate flg22-induced immune responses by positively regulating the interaction between FLS2 and BAK1 (Yeh et al., 2016; Stegmann et al., 2017). Further, FER was reported to regulate the root microbiome composition under phosphate starvation conditions (Tang et al., 2022). Importantly, Arabidopsis ANX1 and soybean GMLMM1 negatively regulate flg22-induced immunity by inhibiting the FLS2-BAK1 interaction (Mang et al., 2017; Wang et al., 2020). In addition to their role in plant PTI, MRLKs function as key regulators of plant ETI. ANX1 and ANX2 negatively regulate NLR protein RPS2-mediated immunity by promoting RPS2 degradation (Mang et al., 2017). Another pair of MRLKs, LET1 and LET2, interact with SUMM2, a CNL protein that monitors the MAPK cascade (Zhang et al., 2012), and positively regulates immunity mediated by SUMM2 (Huang et al., 2020; Liu et al., 2020).

To date, most of the plant MRLKs, especially the non-CrRLK1L-type MRLKs, have not been well studied. In this study, we identified 72 GmMRLKs in soybean plants and found that half of them were non-CrRLK1L type MRLKs. We successfully cloned 60 *GmMRLKs*, transiently expressed them in *N. benthamiana* and systematically analyzed their effects on plant PTI and ETI activation. We identified a number of GmMRLKs that function in plant immunity and revealed the common and specific roles of GmMRLKs in different PTI and ETI immune responses. We showed that most of the identified *GmMRLKs* are negative regulators of plant immunity and are candidate susceptible (S) genes that might be engineered for soybean breeding.

## Materials and Methods

### Bioinformatic Analysis of GmMRLKs

The protein sequences of GmMRLKs were obtained from the Phytozome database^1^ (Glycine max_v2.1, Schmutz et al., 2010), and exhibited in Supplementary Table 1. We searched all the candidate protein sequence of soybean using HMMER 3.1 software with both the PFAM protein files, malectin/malectin-like (PF12819) and pkinase-Tyr (PF07714). Candidate members were submitted on the SMART for manual confirmation of GmMRLK members. ClustalW software was used for multiple sequence alignment. We build phylogenetic trees by MEGA11 using maximum -likelihood method, which was analyzed with 1,000 replicates of bootstrap values. The conserved structural domain analysis of the *GmMLRK* gene family was obtained by Batch SMART tool in TBtools software (TBtools, v1.098669) (Chen et al., 2020; Letunic et al., 2021). The localization of *GmMRLK* genes on soybean chromosomes was collected from Phytozome database and figure was generated by TBtools (Chen et al., 2020). Venn diagrams were generated using TBtools to represent the phenotypic genes of GmMRLKs that overlap in different immune systems.

### Plasmid Construction and Transient Expression in *Nicotiana benthamiana*

For cloning of the *GmMRLKs*, the primers were designed based on the sequences obtained from the Phytozome database^1^. The genomic sequences were amplified from Williasm 82 genomic DNA and inserted them into pCAMBIA1300-35S-HA-RBS. These constructs were introduced into *Agrobacterium* and transiently expressed in *N. benthamiana* for the indicated time. The *N. benthamiana* plants used in this study were grown in plant growth chambers at 25°C with 65% humidity and 12/12 h photoperiod. The primers used in this study was listed in Supplementary Table 2.

### *Phytophthora sojae* and *Pseudomonas syringae* pv. *glycinea* Infection Assay

*Phytophthora sojae* isolate P7076 was cultured on 10% (v/v) V8 juice medium at 25°C in the dark. Soybean cultivar Williams 82 was grown in growth chambers at 25°C with 65% humidity and 16 h photoperiod. The *P. sojae* infestation assay in soybean hypocotyls was performed as previously reported (Song et al., 2015). Briefly, Hypocotyls of etiolated soybean seedlings at 4 days old were inoculated with mycelium blocks of *P. sojae*. The treated hypocotyls were placed under 25°C in the dark. Samples were collected at 0, 24, and 36, for qPCR analysis.

*Phytophthora sojae* infestation of transgenic hairy roots was performed as previously described (Xiong et al., 2014). The *GmMRLKs* transgenic hairy roots were dipped in *P. sojae* zoospore suspension (Concentration of 10^4^ per ml) for 5 mins, and placed on 0.6% agar medium in the dark at 25°C. Samples were stained with Trypan Blue and photographed 36 h later.

*Pseudomonas syringae* pv. *glycinea* (*Psg*) infestation assay in soybean leaves was shown as previously described (Ashfield et al., 1995). Soybean leaves at 14-day-old were infiltrated with bacterium at a concentration of 5 × 10^5^CFU/mL. Samples were taken at 0, 6, 12, and 24 h after infection for qPCR analysis.

### Analysis of *GmMRLK* Gene Expression Patterns

To analyze the expression pattern of *GmMRLK* genes in different tissues and different pathogen (*P. sojae* and *S. sclerotiorum*) infection conditions, the transcriptome data was obtained from the genevestigator database^2^, and the heat map was generated by TBtools (Chen et al., 2020).

### qRT-PCR Assay

The hypocotyls of etiolated soybean seedlings (4-day-old) were inoculated with mycelia of *P. sojae* for 0, 24, and 36 h. Two-week-old soybean plants were inoculated with *Psg* for 0, 6, 12, and 24 h and total RNA was extracted by Trizol reagent (Tsingke, Beijing, China). First-strand cDNA synthesis was performed using M-MLV RNA transcriptase (Takara, Tokyo, Japan), and qPCR was carried out by using specific primers and 2 × RealStar Fast SYBR qPCR Mix (GeneStar, Beijing, China) to detect the expression level of *GmMRLKs* genes. GmELF1β was used as an internal reference gene.

### Luciferase Complementation Image Assay

The Luciferase Complementation Image (LCI) assay was performed according to previous report (Zhou et al., 2018). Shortly, Nluc-HA- and Cluc-tagged proteins were co-expressed in *N. benthamiana* for 2 days using *Agrobacterium*-mediated transient transformation. Leaf disks were taken and incubate in 96-well plates with 1 mM luciferin (BioVision) for 10 mins. Relative luminescence unit (RLU) was detected by a luminometer (TECAN, Männedorf, Switzerland).

### Reactive Oxygen Species Burst Assay

Pathogen-associated molecular pattern (PAMP)-triggered ROS was performed according to the previous reports (Wang et al., 2020). Briefly, the indicated GmMRLKs were expressed in *N. benthamiana* leaves for 2 days by *Agrobacterium*-mediated transient transformation. Leaf disks were taken and incubated overnight with 200 μL of sterile water in a 96-well plate. Leaf disks were treated with luminescence detection mixture containing 1 μM of flg22 (Sangon, Shanghai, China) or 200 μg/mL of chitin (Sigma-Aldrich, St. Louis, MO, United States), 20 mM of luminol (Sigma-Aldrich, St. Louis, MO, United States) and 10 mg/ML of horseradish peroxidase (Sigma-Aldrich, St. Louis, MO, United States), and relative luminescence unit (RLU) was recorded by a luminometer (TECAN, Männedorf, Switzerland).

### Pathogen-Associated Molecular Pattern and Effector-Induced Cell Death Examination

The GmMRLKs were transiently expressed in *N. benthamiana*, and *Agrobacterium* carrying the indicated PAMPs or effectors were infiltrated into *N. benthamiana* leaves. *Agrobacterium*-mediated transient transformation was followed as described (Wang et al., 2011). To analyze the inhibition of PAMPs (XEG1 and INF1) and effectors (AvrB and XopQ)-induced cell death by GmMRLKs, Agrobacterium which carried the each *GmMRLKs* gene was infiltrated into leaves. *Agrobacterium* carried the pCAMBIA1300-GFP empty vector as a control. The same areas were infiltrated with Agrobacterium with XEG1 or INF1 or AvrB or XopQ after 24 h. The cell death phenotype was visualized and photographed under UV light 2–5 days later.

To examine the cell death by electrolyte leakage assay, leaf disks were taken and floated with 5 mL distilled water for 3 h at room temperature, and ion leakage was measured as previously described (Mittler et al., 1999). Briefly, measured with conductivity meter (METTLER TOLEDO, Switzerland) as “value A” after 3 h incubation. Then the samples were boiled for 20 min, allow the solution to cool to room temperature, and the conductivity was measured again to give “value B.” For each sample, ion leakage was exhibited as percent leakage, that is (value A/value B) × 100%. The experiments were repeated three times.

### *A. Rhizogenes*-Mediated Transformation of Soybean Hairy Roots

Soybean seeds were surface sterilized and soaked overnight in sterile water, germinated on medium containing 0.5% sucrose and 1.2% agar and placed in 25°C with a 16 h photoperiod. After 5 days of germination, unblemished cotyledons were harvested and subjected to *Agrobacterium. rhizogenes* (*A. rhizogenes*)-mediated transformation. Transformation assays were carried out as previously described (Yan et al., 2014). Briefly, a roughly circular cut was made in the cotyledon near the end of the petiole, placed on 0.6% agar medium and treated with 20 μl of *A. rhizogenes.* After sealed, the plates were placed in a dark 25°C incubator. Transformed hairy roots grew out on a callus ridge of inoculated cotyledonary 3 weeks later.

## Results

### Identification and Characterization of *GmMRLK*s in Soybean

**FIGURE 1:**
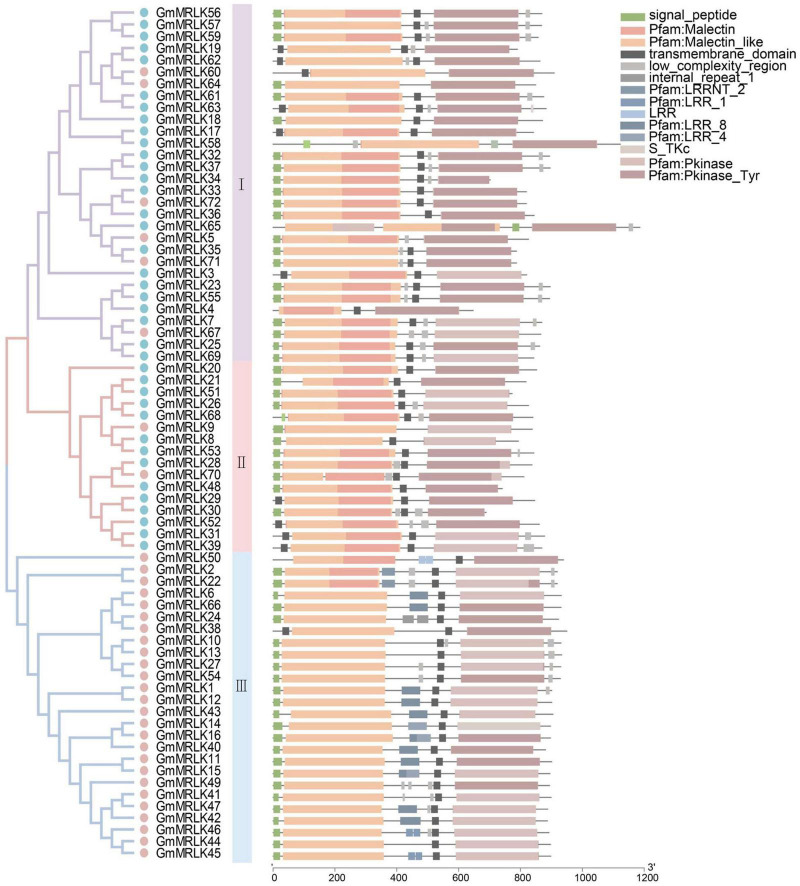
Analyzation of malectin domain-containing receptor-like kinases (MRLKs) in soybean. Phylogenetic tree analysis of soybean MRLKs. The protein sequences of GmMRLKs were aligned by ClustalW. The three different subgroups are marked by shadows of purple, green, and orange colors. The CrRLK1L proteins were marked by blue dots and the remaining GmMRLKs were marked by pink dots. Domain structures of GmMRLKs. The motif composition of the GmMRLKs were analyzed by Batch SMART. Different motifs were represented by rectangles of different colors.

To identify MRLKs in soybeans, we searched the soybean genome for proteins with at least one extracellular malectin-like domain and one intracellular kinase domain. We obtained a total of 72 MRLKs and named them *GmMRLK1*∼*72* according to their positioning on the chromosome (Figure 1 and Supplementary Figure 1). The phylogenetic tree revealed that they could be divided into subgroups I–III (Figure 1), including 38 reported CrRLK1L proteins that fell into subgroup I and II. Subgroup III consisted of 26 members, and all of them were the non-CrRLK1L type. Additionally, the number of GmMRLKs was almost twice that of GmCrRLK1L proteins (Figure 1). Notably, protein domain analysis showed that all GmMRLKs contained an intracellular kinase domain and 1–2 extracellular malectin-like domains (Figure 1). Interestingly, we found that most of subgroup III members contained an extracellular LRR domain in addition to the malectin-like domain. Chromosomal distribution analysis showed that the GmMRLK localized on all the soybean chromosomes. A number of GmMRLKs were tandemly repeated on chromosome 8, 13, 15, and 18 (Supplementary Figure 1). To further analyse the phylogeny of GmMRLKs, we analyzed the distribution of MRLK proteins in *Arabidopsis*, soybean and *Populus trichocarpa* (Kumar et al., 2020). As shown in Supplementary Figure 2, most of the GmMRLKs are tightly grouped with MRLKs in *Arabidopsis* and *Populus trichocarpa* (Supplementary Figure 2).

Next, we analyzed the expression pattern of *GmMRLKs* in different soybean tissues using the Genevestigator database (see text footnote 2). We observed that most *GmMRLKs* showed certain levels of tissue-specific expression (Supplementary Figure 3), indicating that *GmMRLKs* might have tissue-specific functions. For example, the subgroup I *GmMRLKs* were preferentially expressed in cotyledon shoots and leaves, and the subgroup III *GmMRLKs* were highly expressed in roots, root hairs, and pods. Several *GmMRLKs* showed specific high expression in anthers, suggesting that they might participate in reproductive processes (Supplementary Figure 3).

**FIGURE 2:**
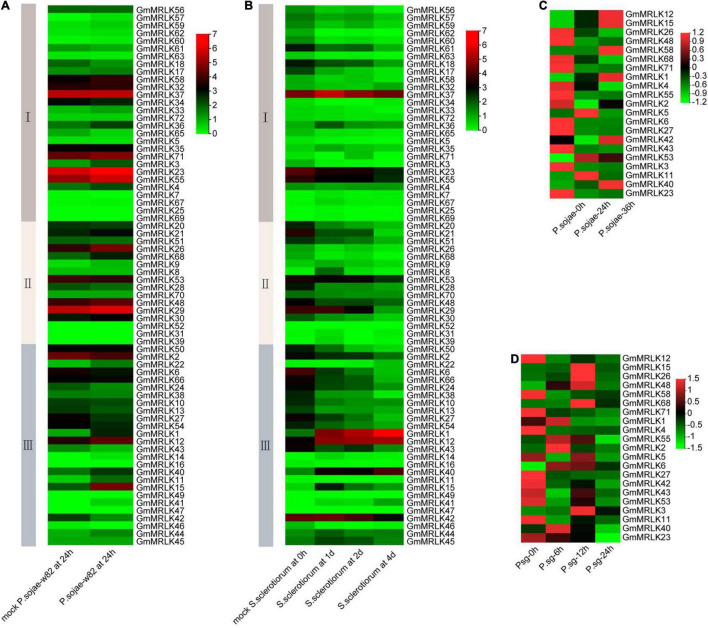
Transcriptional expression of *GmMRLK* genes upon phytopathogens treatment. **(A)** Expression of *GmMRLKs* in response to *P. sojae* for 0 and 24 h. The analyzations were performed by Genevestigator database (https://www.genevestigator.com). The relative expression level of the *GmMRLKs* development stages were showed in the heatmap. **(B)** Expression of *GmMRLKs* in response to *S. sclerotiorum* at different infection stages. The analyzations were performed as in panel **(A)**. **(C)** Examination of *GmMRLKs* expression in responses to *P. sojae* by qPCR analysis. The hypocotyls of etiolated soybean seedlings at 4-days-old were treated with *P. sojae* 0, 24, and 36 h, total RNA was extracted and expression of the indicated *GmMRLKs* were analyzed by qPCR and showed by heatmap. **(D)** Examination of *GmMRLKs* expression in responses to *Pseudomonas syringae* pv. *glycinea* (*Psg*) by qPCR analysis. Soybean plants at 2-week-old were treated with *P. sojae* 0, 6, 12, and 24 h, total RNA was extracted and expression of the indicated *GmMRLKs* were analyzed by qPCR and showed by heatmap.

To preliminarily determine the roles of GmMRLKs in plant immunity and resistance, we analyzed the expression patterns of *GmMRLKs* in response to different phytopathogens. The results showed that 14 *GmMRLKs* were transcriptional induced whereas 7 *GmMRLKs* were downregulated by *P. sojae* treatment at 24 h (Figure 2A). During the *S. sclerotiorum* infection process, we noticed that the *GmMRLK*s were differentially induced or suppressed at different days post-incubation (Figure 2B), suggesting that individual GmMRLKs function during different infection time periods. *P. sojae* infection is a devastating disease that causes significant losses in soybean production (Tyler, 2007). Thus, we selected 21 differentially expressed *GmMRLKs* upon *P. sojae* infection for qPCR verification. We treated soybean plants with *P. sojae* infection for 0, 24, and 36 h, and examined the expression of the indicated *GmMRLKs*. We observed that 8 *GmMRLKs* (*GmMRLK1, 5, 11, 12*, *15*, *23*, *40*, *53*, *58*) were induced and 11 *GmMRLKs* (*GmMRLK2, 3, 6, 23, 26*, *27*, *42*, *43*, *48*, *55*, *71*) were downregulate upon *P. sojae* treatment at 24 h (Figure 2C and Supplementary Figure 4). *GmMRLK5* and *GmMRLK11* were induced at 24 h, but showed compromised expression after 36 h treatment (Figure 2C and Supplementary Figure 4). Next, we treated the soybean plants with *Psg*, a causative bacterial pathogen that caused leaf spot in soybean, and examined the expression of 21 differentially expressed *GmMRLKs.* We showed most of the *GmMRLKs* are differentially expressed upon *Psg* treatment (Figure 2D and Supplementary Figure 5). We observed that 11 *GmMRLKs* (GmMRLK2, 3, 6, 11, 15, 26, 27, 40, 48, 55, 68) were induced and 10 *GmMRLKs* (GmMRLK1, 4, 5, 12, 23, 42, 43, 53, 58, 71) were downregulate upon *Psg* treatment. These results indicate that these *GmMRLKs* may be involved in soybean immunity at an early stage.

### Identification of *GmMRLKs* Involved in Pathogen-Associated Molecular Pattern-Triggered Reactive Oxygen Species Production

**FIGURE 3:**
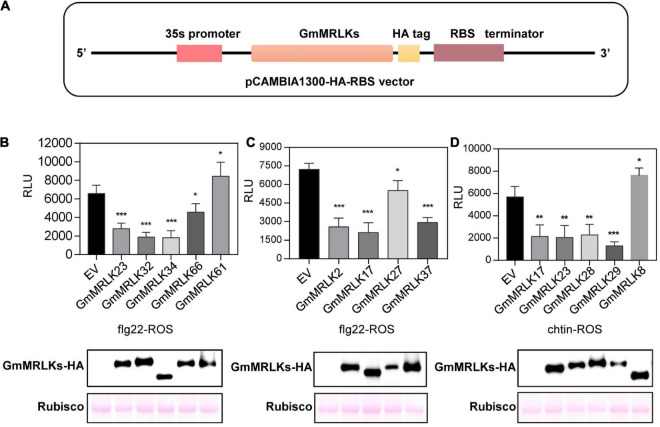
GmMRLKs negatively regulate flg22- and chitin-induced ROS production in *N. benthamiana* plants. **(A)** Cloning of the *GmMRLKs*. The genomic sequences of *GmMRLKs* were cloned into the pCAMBIA1300-35S-HA-RBS vector, which were driven by 35S promoter, and followed by a HA tag and RBS terminator. **(B,C)** Effect of GmMRLKs on flg22-induced ROS burst. The indicated GmMRLKs were expressed in *N. benthamiana* plants by *Agrobacterium*-mediated transient expression for 2 days, and subjected to flg22-induced ROS examination. EV, empty vector. (Mean ± SD, *n* ≥ 6, Student’s *t*-test; *p < 0.05, ***p < 0.001). **(D)** Effect of GmMRLKs on chitin-induced ROS burst. The indicated GmMRLKs were expressed in *N. benthamiana* plants by *Agrobacterium*-mediated transient expression for 2 days, and subjected to chitin-induced ROS examination (mean ± SD, *n* ≥ 6, student’s *t*-test; **p < 0.01).

To analyze the role of GmMRLKs in plant immunity, we amplified 60 *GmMRLK* genes from Williams 82 genomic DNA and inserted them into the pCAMBIA1300-35S-HA-RBS vector (Figure 3A and Supplementary Table 3). In the vector, *GmMRLKs* were driven by the 35S promoter and fused with a C-terminal HA tag. We sought to analyze the function of GmMRLKs in plant immunity using an *Agrobacterium*-mediated transient transformation system in *N. benthamiana* leaves. PAMPs-induced transient ROS burst is a specific assay for examination of PTI activation. Thus, we transiently expressed GmMRLKs in *N. benthamiana* for 2 days and examined the ROS burst induced by bacterial flg22 and fungal chitin. Notably, the expression of GmMRLK1, GmMRLK3, GmMRLK5, GmMRLK7, and GmMRLK12 caused obvious cell death phenotypes in *N. benthamiana* (Supplementary Figures 6A,B). Thus, these 5 GmMRLKs were not applied to the ROS examination assay.

We observed that GmMRLK2, 17, 23, 27, 32, 34, 37, and 66 significantly suppressed flg22-induced ROS production (Figures 3B,C). GmMRLK37 was previously reported to be GmLMM1, which negatively regulates plant immunity (Wang et al., 2020). We observed that flg22-induced ROS was slightly but significantly enhanced by the expression of GmGMRLK61 (Figure 3B), indicating that it might play a positive role in PTI activation. Chitin-induced ROS production was severely suppressed by GmMRLK 17, 23, 28, and 29, but was enhanced by GmMRLK8 (Figure 3D). Immunoblot blot assays showed that these GmMRLK proteins were normally expressed in *N. benthamiana* plants (Figures 3B–D). The remaining GmMRLKs had no effect on flg22- and chitin-induced ROS bursts (Supplementary Figure 7).

### Identification of GmMRLKs Involved in Pathogen-Associated Molecular Pattern-Induced Cell Death

**FIGURE 4:**
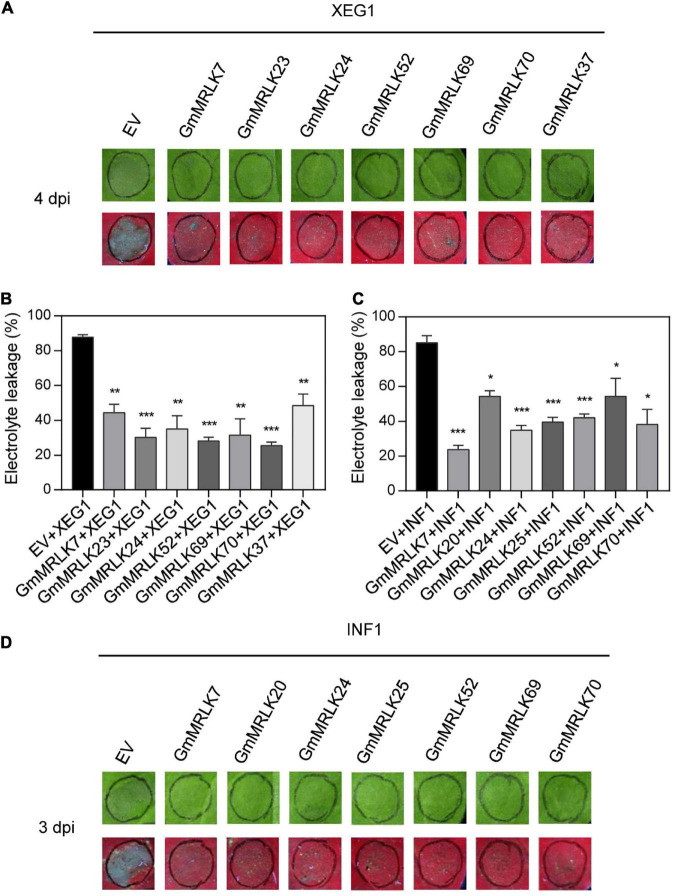
GmMRLKs suppress XEG1- and INF1-induced cell death. **(A,B)** GmMRLKs suppress *P. sojae*-derived PAMP XEG1-induced cell death. The indicated GmMRLKs were transiently expressed in *N. benthamiana* for 1 day, and infiltrated with *Agrobacterium* carrying XEG1. The cell death phenotype was examined by taking photograph under UV light **(A)** and measuring the electrolyte leakage **(B)**. (Mean ± SD, *n* ≥ 8, student’s *t*-test; **p < 0.01, ***p < 0.001). **(C,D)** GmMRLKs suppress *P. infestans*-derived PAMP INF1-induced cell death. The indicated GmMRLKs were transiently expressed in *N. benthamiana* for 1 day, and infiltrated with *Agrobacterium* carrying INF1. The cell death phenotype was examined by taking photograph under UV light **(D)** and measuring the electrolyte leakage **(C)**. (Mean ± SD, *n* ≥ 8, student’s *t*-test; *p < 0.05, ***p < 0.001).

To further analyze the role of GmMRLKs in PTI responses, we examined the effect of GmMRLKs on cell death induced by PAMPs including *P. sojae*-derived XEG1 and *P. infense*-derived INF1. The GmMRLKs were transiently expressed in *N. benthamiana* leaves 1 day before infiltration of *Agrobacterium* carrying XEG1 or INF1. As shown in Figure 4A, XEG1-induced cell death was significantly suppressed by the expression of GmMRLK7, 23, 24, 52, 69, 70 and GmMRLK37 (GmLMM1) (Figure 4A). PAMP-induced cell death caused ion leakage from cells, which in turn changes the conductivity of plant tissues. Thus, we performed the ion leakage experiment to measure the conductivity and confirmed the cell death phenotypes shown in Figure 4B. We next examined INF-induced cell death by the cell death visualization and ion leakage experiments. The results showed that INF1-induced cell death was significantly suppressed by the expression of GmMRLK7, 20, 24, 25, 32, 69, and 70 (Figures 4C,D). The remaining GmMRLKs had no effect on XEG1- or INF1-induced cell death (Supplementary Figure 7).

### Identification of GmMRLKs Involved in Effector-Induced Cell Death

**FIGURE 5:**
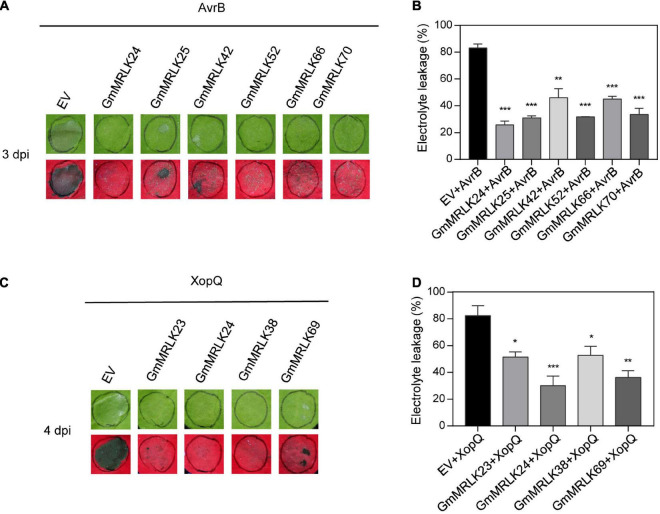
GmMRLKs suppress AvrB and XopQ-induced HR reaction. **(A,B)** GmMRLKs suppress *Pseudomonas syringae* effector AvrB -induced HR. The indicated GmMRLKs were transiently expressed in *N. benthamiana* for 1 day, and infiltrated with *Agrobacterium* carrying AvrB. The cell death phenotype was examined by taking photograph under UV light **(A)** and measuring the electrolyte leakage **(B)**. (Mean ± SD, *n* ≥ 8, student’s *t*-test; **p < 0.01, ***p < 0.001). **(C,D)** GmMRLKs suppress *Xanthomonas campestris* effector XopQ-induced HR. The indicated GmMRLKs were transiently expressed in *N. benthamiana* for 1 day, and infiltrated with *Agrobacterium* carrying XopQ. The cell death phenotype was examined by taking photograph under UV light **(C)** and measuring the electrolyte leakage **(D)**. (Mean ± SD, *n* ≥ 8, student’s *t*-test; *p < 0.05, **p < 0.01, ***p < 0.001).

We next sought to analyze the role of GmMLRKs in plant ETI. The hypersensitive response (HR) triggered by microbial effectors is a hallmark of plant ETI activation. *Pseudmonas syringae*-derived AvrB and *Xanthomonas campestris*-derived XopQ induce cell death (i.e., HR HR) in *N. benthamiana* plants, and they are recognized by CNL and TNL, respectively (Bisgrove et al., 1994; Grant et al., 1995; Jones et al., 2016; Schultink et al., 2017). We noticed that AvrB-induced cell death was severely impaired by expression of GmMRLK24, 25, 42, 52, 66, and 70 (Figure 5A). Whereas, XopQ-triggered cell death was suppressed by GmMRLK23, 24, 38, and 69 (Figure 5C). All AvrB/XopQ-induced cell death phenotypes were confirmed by ion leakage experiments by measuring the conductivity (Figures 5B,D). None of the GmMRLKs exhibited enhanced AvrB/XopQ-induced HR responses (Supplementary Figure 7).

### Summary of the Roles of GmMRLK in Plant Immunity

**FIGURE 6:**
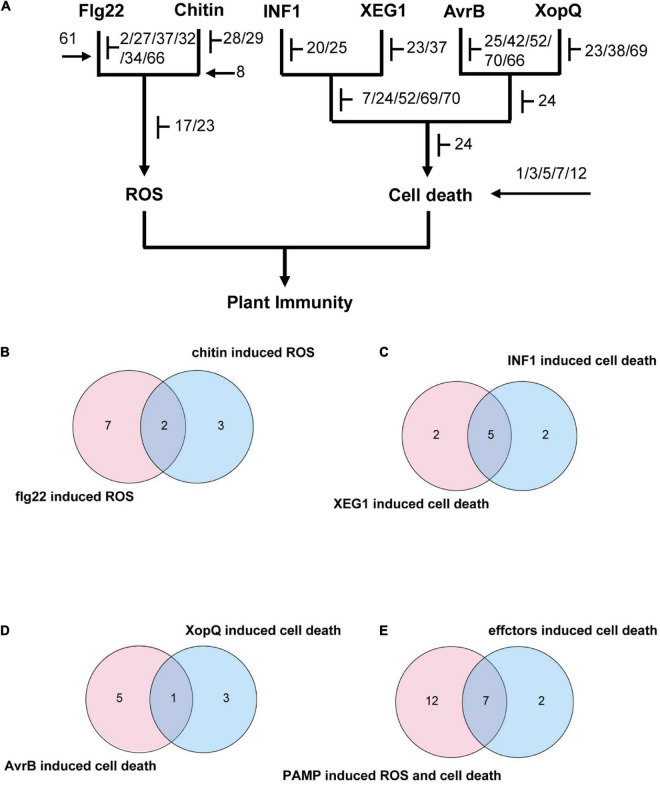
Summary the roles of GmMRLKs in different plant immune responses. **(A)** Classification of GmMRLKs based on their effects on different immune responses. *T* bar indicates suppression of immunity and arrows indicates promotion of immunity. In the chart, GmMRLKs that can be connected to certain immune response indicating that they play a role in this immune response. **(B)** Venn diagram showing the number of GmMRLKs that function in flg22- and chitin-induced ROS production. **(C)** Venn diagram showing the number of GmMRLKs that function in XEG1- and INF1-induced cell death. **(D)** Venn diagram showing the number of GmMRLKs that function in AvrB- and XopQ-induced HR. **(E)** Venn diagram showing the number of GmMRLKs that function in PTI and ETI.

Based on the plant immunity assays performed above, we summarized the results of all the PTI-and ETI-related assays, including flg22- and chitin-induced ROS burst, XEG1- and INF-induced cell death, and AvrB- and XopQ-induced HR reactions. All the results were exhibited by heatmap in Supplementary Figure 7. Based on these results, we further analyzed the common and specific roles of GmMRLKs in immunity mediated by different PRRs and NLRs. We classified GmMRLKs based on their effect on different immune responses and visually displayed functional specificity and redundancy in a diagram (Figure 6A). We showed that 9 GmMRLKs function in flg22-induced ROS and 5 GmMRLKs function in chitin-induced ROS. GmMRLK17 and GmMRLK23 were involved in both flg22- and chitin-induced ROS bursts (Figure 6B). In the PAMP-induced cell death assay, we observed that GmMRLK7, 24, 52, 69, and 70 were involved in both XEG1- and INF1-induced cell death. While GmMRLK23 and GmMRLK37 specifically function in XEG1-induced cell death, GmMRLK20 and GmMRLK25 specifically regulate INF1-induced cell death (Figure 6C). We showed that 6 and 4 GmMRLKs function in AvrB- and XopQ-induced HR reactions, respectively. GmMRLK24 overlapped between the two groups, functioning in both avrB- and XopQ-induced ETI activation (Figure 6D).

Finally, we summarized the GmMRLKs that function in PTI (PAMP-induced ROS and cell death) and ETI (effector-induced HR). We showed that 19 and 9 GmMRLKs function in PTI and ETI, respectively (Figure 6E). Among them, 7 GmMRLKs (GmMRLK23, 24, 25, 52, 66, 69, and 70) overlapped (Figure 6E), indicating that these genes regulate both PTI and ETI and might be involved in PTI-ETI crosstalk.

### GmMRLKs Regulate Plant Immunity Probably by Directly Coupling to the Cell Surface and Intracellular Immune Receptor Complexes

**FIGURE 7:**
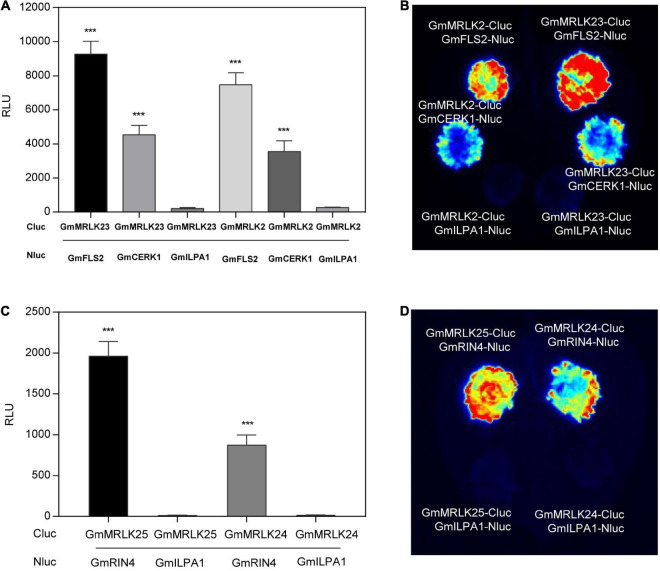
GmMRLKs are probably involved in plant immune receptor complexes. **(A,B)** GmMRLK2 and GmMRLK23 interaction with GmFLS2 and GmCERK1. The indicated Nluc and Cluc constructs were expressed in *N. benthamiana* by *Agrobacterium*-mediated transient expression for 2 days, and subjected to luciferase complementation image (LCI) assays. The protein interaction intensity was showed by measuring relative luminescence unit (RLU) using luminometer **(A)** or by CCD imaging **(B)**. GmILPA1 was used as a negative control. (Mean ± SD, *n* ≥ 8, student’s *t*-test; ***p < 0.001). **(C,D)** GmMRLK24 and GmMRLK25 interaction with GmRIN4. The indicated Nluc and Cluc constructs were expressed in *N. benthamiana* by *Agrobacterium*-mediated transient expression for 2 days, and subjected to LCI assays. The protein interaction intensity was showed by measuring RLU using luminometer **(C)** or by CCD imaging **(D)**. GmILPA1 was used as a negative control. (Mean ± SD, *n* ≥ 8, student’s *t*-test; ***p < 0.001).

Considering the roles of GmMRLKs in plant PTI and ETI, we reasoned that whether GmMRLKs regulate plant immunity by directly coupling with plant PRR or NLR receptor complexes. To verify this notion, we selected GmMRLK23 and GmMRLK2, which function in PTI activation, and examined their interaction with GmFLS2 and GmCERK1 using luciferase complementation image (LCI) assays. Both GmMRLKs showed strong interactions with GmFLS2 and GmCERK1 (Figures 7A,B), indicating that GmMRLKs are components of the plant PRR receptor complexes. GmILPA1-Nluc was used as a negative control.

We next selected GmMRLK24 and GmMRLK25, two GmMRLKs that negatively regulate AvrB-induced HR, to examine their interaction with GmRIN4a, which is essential for AvrB recognition and form a complex with AvrB and the NLR RPG1-B (Selote and Kachroo, 2010; Selote et al., 2013). The LCI assay showed that GmMRLK24 and GmMRLK25 exhibited strong interactions with GmRIN4, but not with GmILPA1 (Figures 7C,D). Thus, we reasoned that GmMRLKs might be involved in the NLR receptor complexes. Taken together, these results indicate that GmMRLKs regulate plant immunity probably by interacting with plant cell-surface PRRs and intracellular NLR receptor complexes.

### GmMRLK7 and GmMRLK25 Significantly Enhanced Soybean Resistance to *Phytophthora sojae* Infection

The aforementioned results showed that most of the identified GmMRLKs were negative regulators of plant immunity. These GmMRLKs can be considered candidate susceptible (S) genes, which can be engineered to improve plant resistance using biotechnologies, such as gene editing. Therefore, we selected GmMRLK7 (inhibited XEG1/INF1-induced cell death), GmMRLK20 (suppressed INF1-induced cell death), and GmMRLK25 (repressed cell death induced by INF1 and AvrB) to examine their roles in soybean resistance to *P. sojae*. We transiently expressed these three *GmMRLKs* and *EV* in soybean hairy roots for 25 days, and inoculated them with *P. sojae* for 36 h. While expression of GmMRLK25 and GmMRLK7 in hairy roots greatly promoted plant susceptibility to *P. sojae*, expression of GmMRLK20 slightly reduced resistance to *P. sojae* (Supplementary Figure 8). Thus, the identified GmMRLKs may play a negative role in plant resistance and are candidate S genes.

## Discussion

For the past years, the CrRLK1L kinases have been extensively studied in model plant Arabidopsis. However, most of the MRLKs in soybean have not been well studied, especially the non-CrRLK1 type GmMRLKs. In this study, we identified 72 MRLKs in soybean containing 34 non-CrRLK1 type GmMRLKs. We successfully cloned 60 *GmMRLKs* and systematically characterized their common and specific roles in plant PTI and ETI mostly based on the transient expression assay in *N. benthamiana* plants, and showed a total number of 21 GmMRLKs are involved in plant immunity.

We examined the effects of GmMRLKs on flg22- and chitin-induced ROS bursts, a typical assay for assessment of PTI activation. We noticed that 9 GmMRLKs were involved in flg22-induced ROS, including GmLMM1 (Wang et al., 2020), and 5 GmMRLKs participated in chitin-induced ROS. These results indicated that GmMRLKs regulate different PAMP-induced immunity with redundancy and specificity. It worth noting that 2 GmMRLKs are involved in both flg22- and chitin-triggered ROS. Whether these GmMRLKs are common regulators in PTI and how they regulate different PRR-mediated immunity still need further studies.

Protein-protein interaction assays showed that GmMRLKs interact with GmFLS2 and GmCERK1. In view of these results, we hypothesized that GmMRLKs may function downstream of different PRRs to regulate the PAMP-induced ROS bursts. While most identified GmMRLKs played a negative role in flg22- or chitin-induced ROS bursts. GmMRLK61 positively influenced flg22-induced ROS, and GmMRLK8 positively regulated chitin-induced ROS. These results indicated that GmMRLKs may opposingly affect plant PTI activation. In Arabidopsis, the MRLKs FER, IOS1, and ANX1 play opposing roles in flg22-induced immunity by directly coupling to the FLS2-BAK1 complex (Yeh et al., 2016; Mang et al., 2017; Stegmann et al., 2017). While FER and IOS1 promoted flg22-induced FLS2-BAK1 interaction, ANX1 negatively regulated FLS2-BAK1 complex formation (Yeh et al., 2016; Mang et al., 2017; Stegmann et al., 2017). We have previously reported that soybean MRLK GmLMM1 negatively regulates immunity by suppressing the flg22-induced GmFLS2-GmBAK1 interaction (Wang et al., 2020). Whether the other identified GmMRLKs function through a similar mechanism remains elucidated.

We also examined the effect of GmMRLKs on INF1 and XEG1-triggered cell death. While 7 GmMRLKs suppressed XEG1-induced cell death, another 7 GmMRLKs negatively regulated INF1-induced cell death. A number of 5 GmMRLKs were shared between the two groups, indicating that GmMRLKs play negative roles in PAMP-induced cell death. XEG1 and INF1 are recognized by RXEG1 and ELR receptors, respectively (Du et al., 2015; Wang et al., 2018), both of which are RLP proteins. We observed that the 2 GmRMLKs were involved in both flg22/chitin-induced ROS and XEG1/INF1-induced cell death. In view of these observations, we reasoned that GmMRLKs may function in RLK- and RLP-mediated immunity with redundancy and specificity. It will also be interesting to examined the function specificity of GmMLRKs in immunity mediated by RLP and RLK receptors.

To determine the role of GmMRLKs in ETI, we examined the effects of GmMRLKs on AvrB- and XopQ-induced HR. We noticed that 6 and 4 GmMRLKs function in AvrB and XopQ-induced HR reactions, respectively. Only GmMRLK24 was involved in both AvrB- and XopQ-induced ETI. Therefore, we hypothesized that GmMRLKs were involved in both TNL- and CNL-mediated ETI activation. ANX1 and ANX2, a pair of Arabidopsis MRLKs, have been reported to interact with the NLR protein RPS2 and promote its degradation to negatively regulate ETI activation (Mang et al., 2017). Another pair of MRLKs, LET1 and LET2, interact with the NLR protein SUMM2 and positively regulate SUMM2-mediated immunity (Huang et al., 2020; Liu et al., 2020). Consistent with this, we showed that 3 GmMRLKs, namely, GmMRLK2, 24, and 25, function in AvrB-induced HR, directly interact with RIN4, a guardee protein that is required for AvrB recognition, and directly interacts with the CNL RPG1B and RPM1 (Mackey et al., 2002; Selote and Kachroo, 2010; Liu T. et al., 2011). These results indicated that these GmMRLKs regulate ETI probably by coupling to the NLR complexes. Notably, Our results showed that GmMRLKs might regulate CNL- and TNL-mediated ETI with redundancy and specificity. It will be interesting to further the roles and mechanisms of GmMRLKs in different NLR-mediated immunity.

In our study, we were not able to identify GmMRLKs that positively regulate PAMP or effector-induced cell death. Considering that the PAMPs and effectors used in this study trigger strong cell death, it was difficult to visualize an enhanced cell death phenotype. Thus, we cannot exclude the possibility that some GmMRLKs positively regulate PAMP or effector-induced cell death. Notably, we observed that 5 GmMRLKs triggered cell death. It would be interesting to investigate whether and how these GmMRLKs are involved in ETI in future studies.

Recent studies have shown that PTI and ETI share similar machineries and pathways (Pruitt et al., 2021; Tian et al., 2021), and ETI activates immunity by augmenting PTI (Ngou et al., 2021; Yuan et al., 2021). For example, some MRLKs, such as ANX1 and ANX2, have been reported to regulate both PTI and ETI activation (Mang et al., 2017). Our results showed that 7 GmMRLKs (GmMRLK23,24, 25, 52, 66, 69, and 70) participated in both PTI and ETI. Thus, it is likely that these GmMRLKs may be involved in PTI-ETI crosstalk. Future studies should address this interesting possibility, including testing the specific roles of these GmMRLKs in PTI-ETI crosstalk. Many MRLKs are involved in the recognition of RALF peptides to regulate multiple biological processes including plant immunity (Franck et al., 2018). Future studies should determine whether and how these identified GmMRLKs regulate plant PTI and ETI in coordination with their corresponding RLAFs.

Most of the identified GmMRLKs are negative regulators of plant immunity, and we confirmed this by transient expression of GmMRLK7 and GmMRLK25 in soybean hairy roots, which caused enhanced susceptibility to *P. sojae* infection. Thus, we reasoned that these *GmMRLKs* are candidate susceptibility (S) genes that could be engineered for improvement of soybean resistance. In addition, GmMRLKs are also reported to regulate plant growth, development and reproduction processes (Franck et al., 2018). Future studies need to investigate the role of GmMRLKs in growth and development, particularly in agronomic traits. Uncovering the role and mechanisms of GmMRLKs in balancing immunity and development will further facilitate the breeding of soybean varieties with high resistance and low yield penalties.

## Data Availability Statement

The original contributions presented in this study are included in the article/Supplementary Material, further inquiries can be directed to the corresponding authors.

## Author Contributions

QZ and SC performed most of the experiments. DW, WW, RC, and YL contributed gene cloning and construction. YB contributed to bioinformatic analysis. GX contributed to proteins interaction assays. XF, XL, and DD coordinated the research and wrote the manuscript. All authors contributed to the article and approved the submitted version.

## Conflict of Interest

The authors declare that the research was conducted in the absence of any commercial or financial relationships that could be construed as a potential conflict of interest.

## Publisher’s Note

All claims expressed in this article are solely those of the authors and do not necessarily represent those of their affiliated organizations, or those of the publisher, the editors and the reviewers. Any product that may be evaluated in this article, or claim that may be made by its manufacturer, is not guaranteed or endorsed by the publisher.

## Footnotes

1
https://phytozome-next.jgi.doe.gov/

2
https://genevestigator.com/
