# Supplementary material for: Functional Diversification Analysis of Soybean Malectin/Malectin-Like Domain-Containing Receptor-Like Kinases in Immunity by Transient Expression Assays
Source: Front Plant Sci. 2022 Jun 23;13:938876. doi: 10.3389/fpls.2022.938876 (PMC9260666; doi:10.3389/fpls.2022.938876)
Supplement: Supplementary file 1 [file Data_Sheet_1.docx]

**Supplementary Information**

Functional diversification analysis of soybean malectin/malectin-like domain-

containing receptor-like kinases in immunity by transiently expression assays

Qian Zhang^1^, Shuxian Chen^1^, Yazhou Bao^1^, Dongmei Wang^2^, Weijie Wang^1^, Rubin Chen^1^, Yixin Li^1^, Guangyuan Xu^1^, Xianzhong Feng^2^, Xiangxiu Liang*^1,4^, Daolong Dou*^1,3^


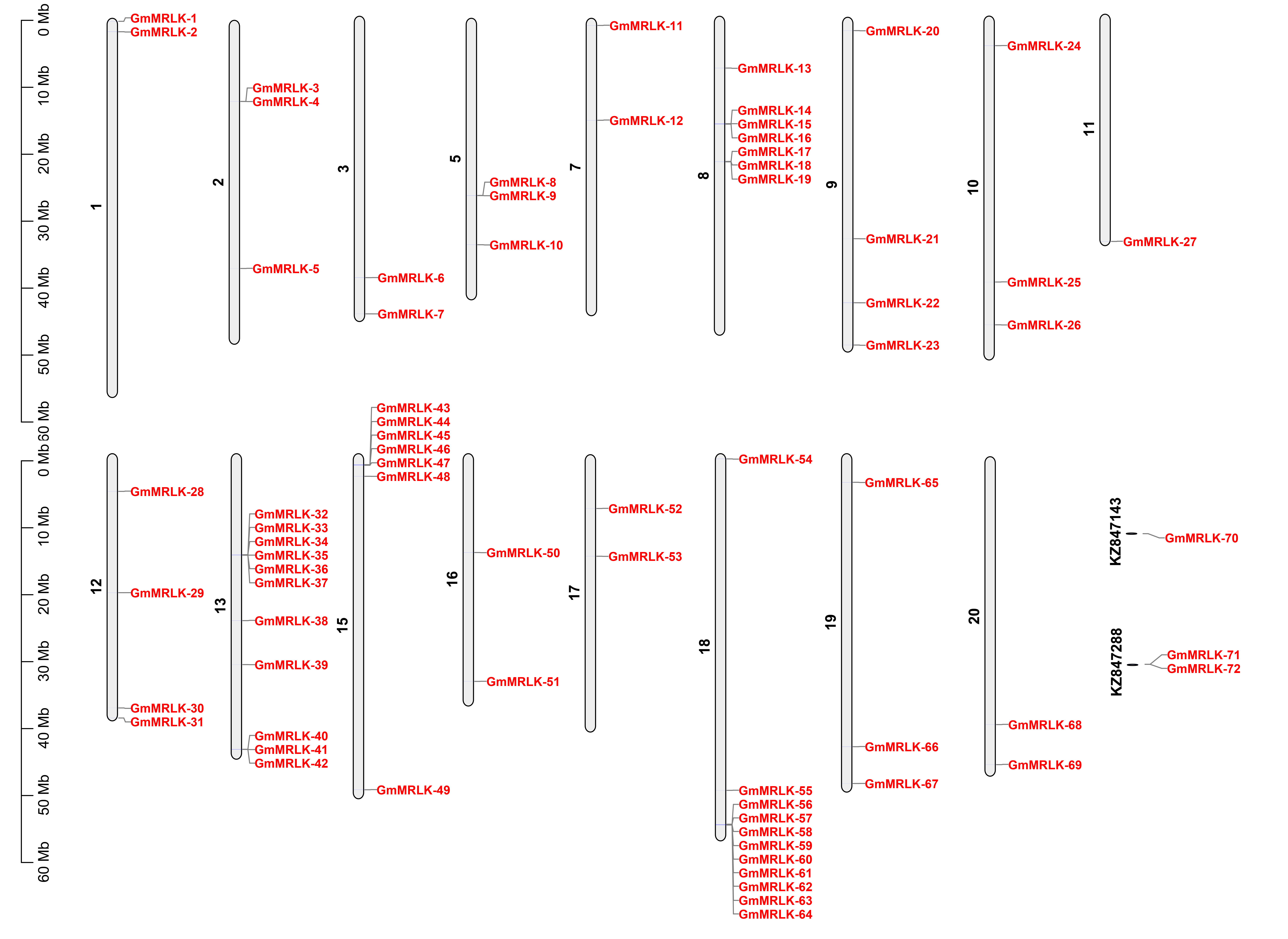


**Figure S1 Chromosomal distribution of *MRLK* genes.**

The chromosome distribution map was created with TBtools software. The ticks on the left are in megabytes.

**
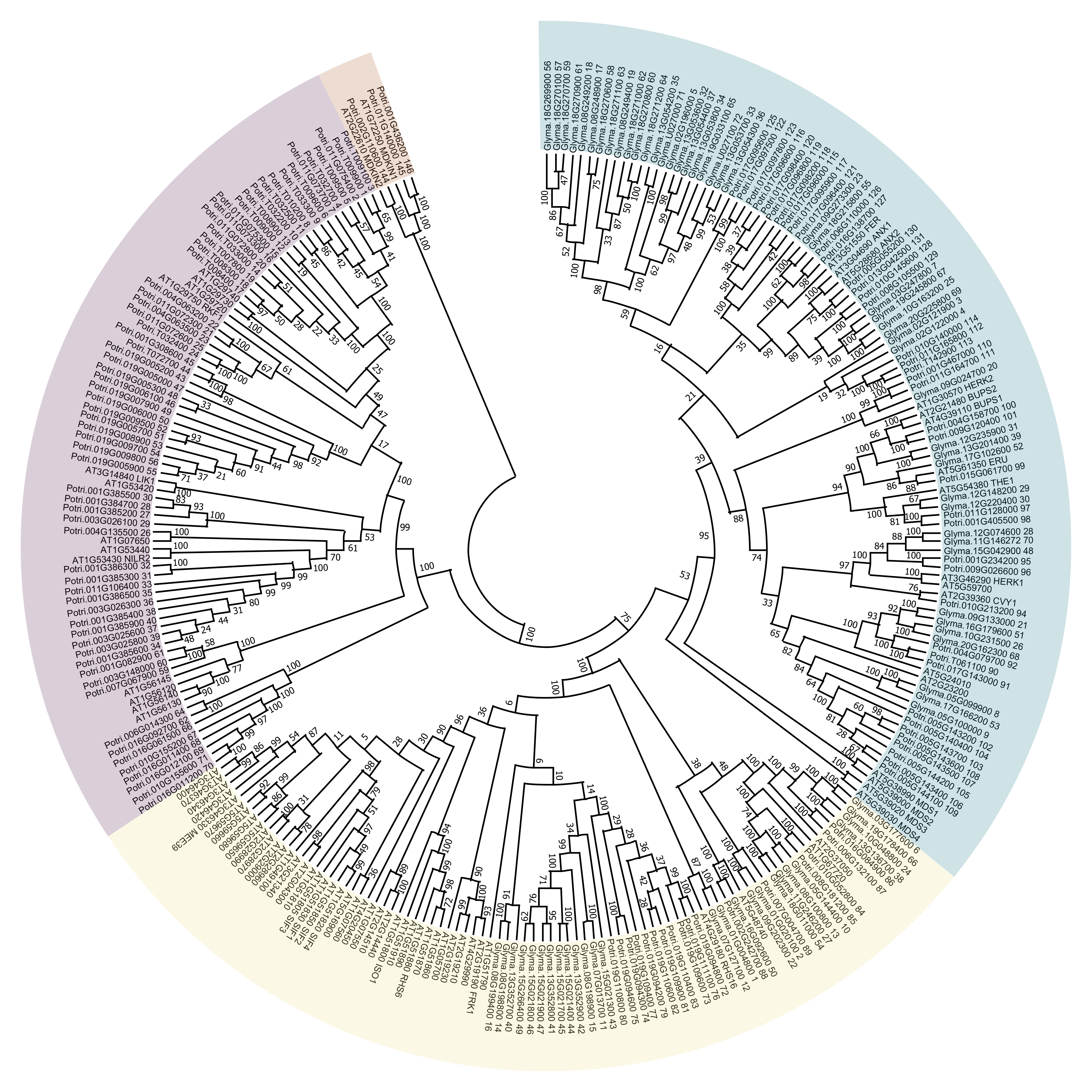
**

**Figure S2 Phylogenetic tree of MRLKs proteins in *Arabidopsis*, soybean, and *Populus trichocarpa*.**

Full length protein sequences of GmMRLKs were ClustalW-aligned to generate the tree by neighbor-joining method using MEGA 11 software.


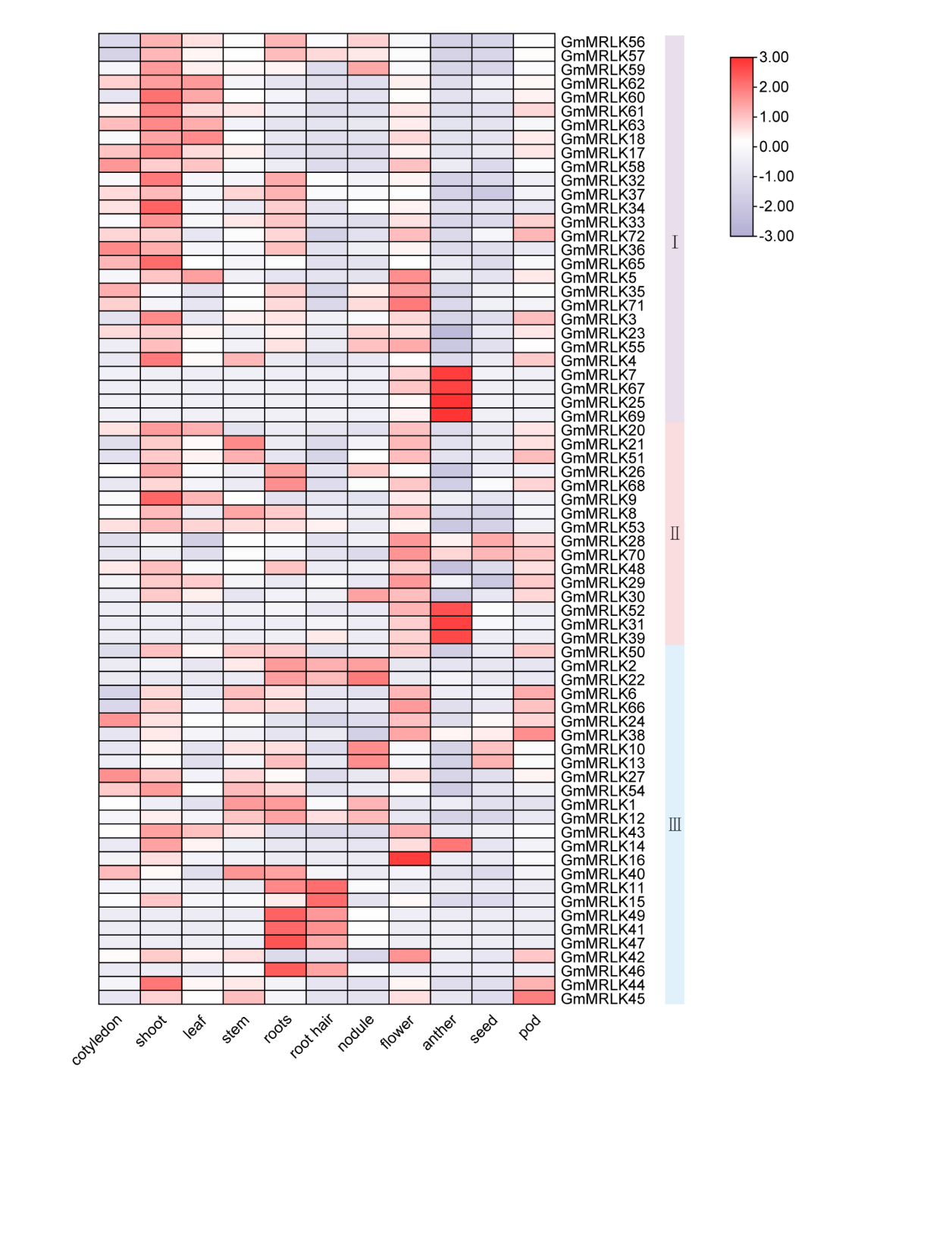


**Figure S3 The expression pattern of GmMRLKs genes in different tissues.**

The relative expression level of the *GmMRLK* genes in different tissues were obtained from Genevestigator database (https://www.genevestigator.com). The heatmap was generated by TBtools (Chen et al., 2020).


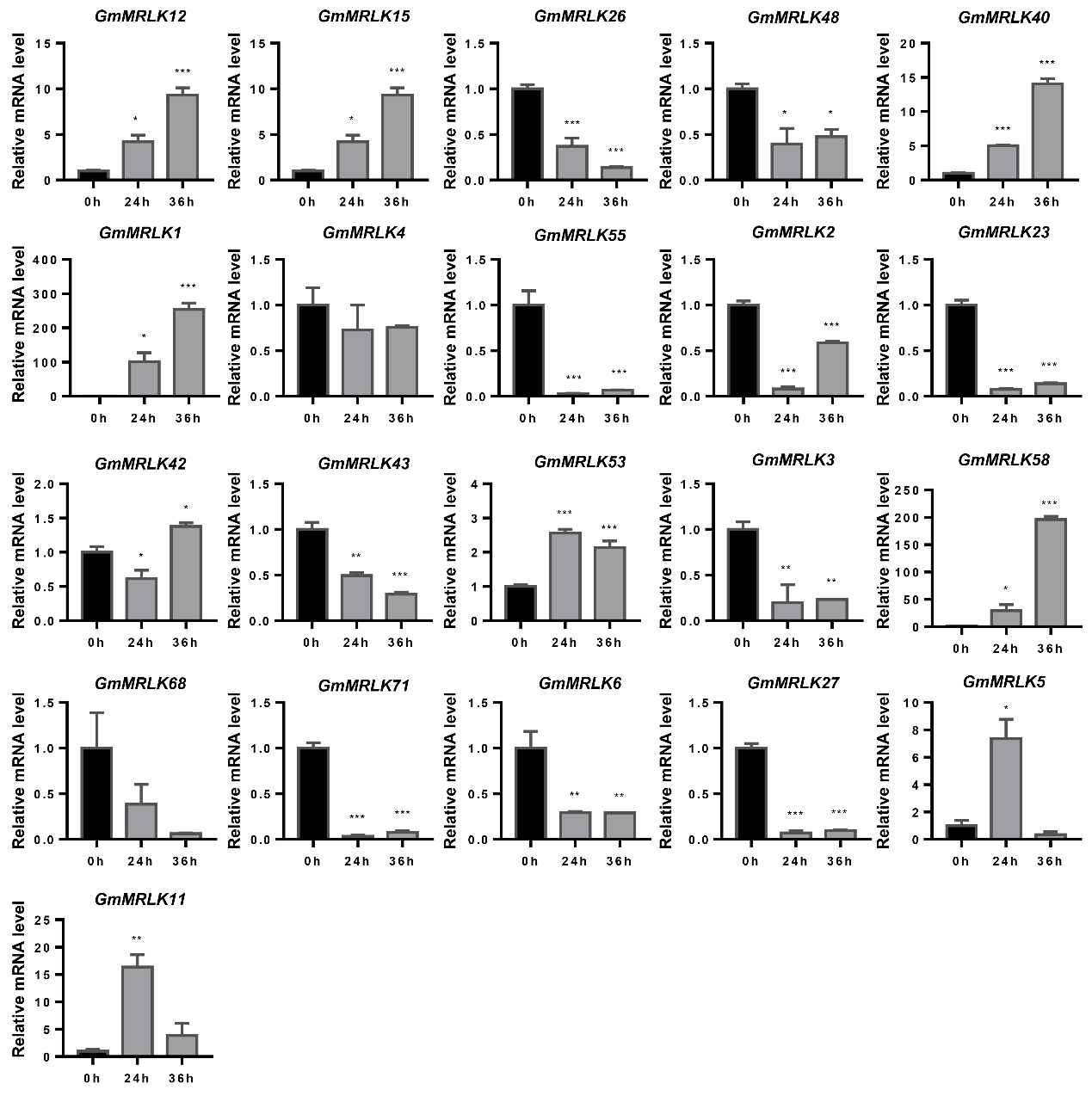


**Figure S4 Expression of *GmMRLK*s in response to *P. sojae* treatment.**

Related to Figure 2C. Soybean plants at four-days-old were treated with *P. sojae* 0, 24 and 36 hrs, total RNA was extracted and expression of the indicated *GmMRLKs* were analyzed by qPCR (Mean± SD; Student’s t-test; *, *p*< 0.05, **, *p*< 0.01, ***, *p*< 0.001).


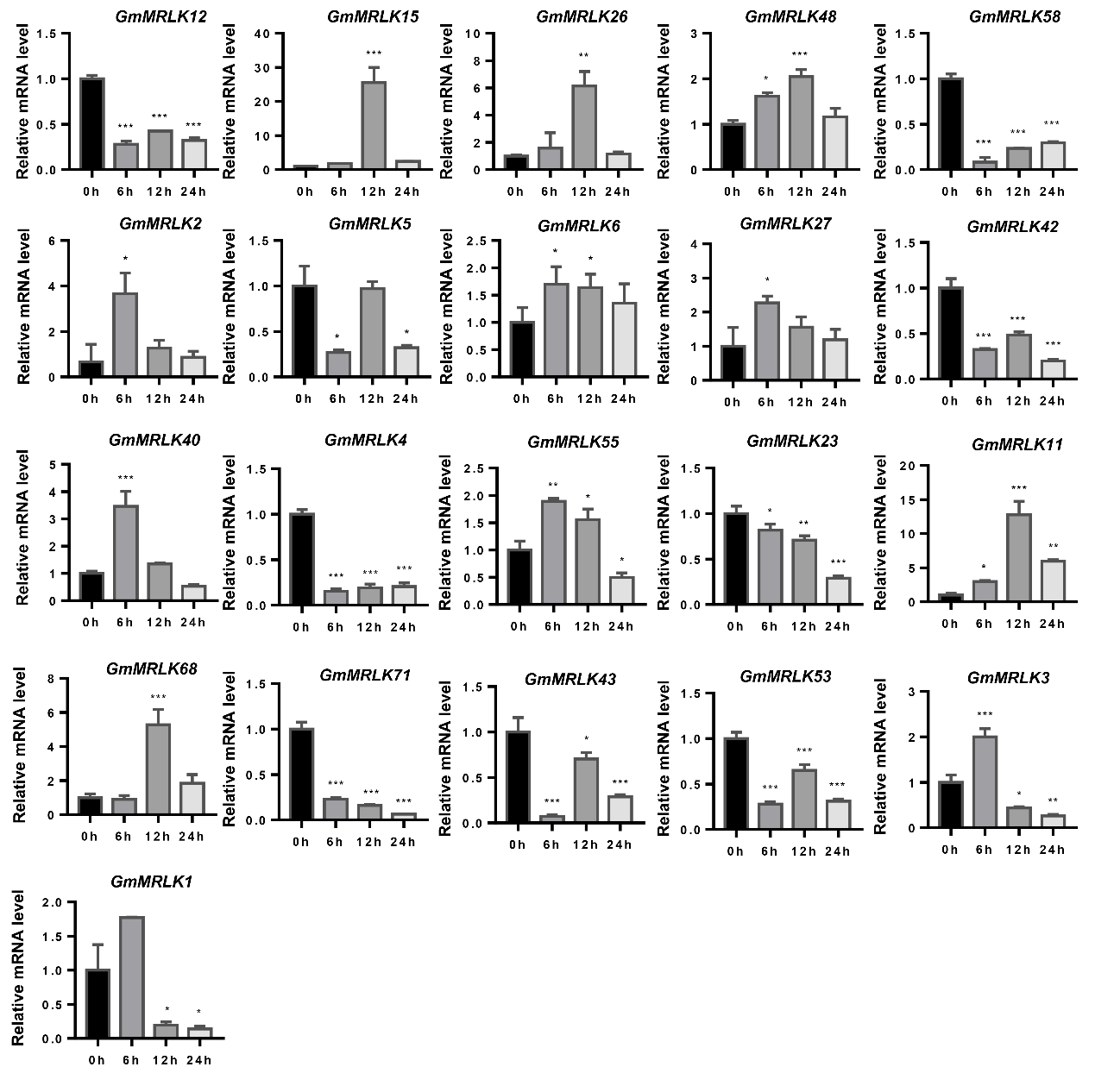


**Figure S5 | Expression of *GmMRLK*s in response to *Pseudomonas syringae* pv. *glycinea* (*Psg*) treatment.**

Related to Figure 2D. Examination of *GmMRLKs* expression in responses to *Pseudomonas syringae* pv. *glycinea* (*Psg*) by qPCR analysis. Soybean plants at two-week-old were treated with *P. sojae* 0, 6,12 and 24 hrs, total RNA was extracted and expression of the indicated *GmMRLKs* were analyzed by qPCR (Mean± SD; Student’s t-test; *, *p*< 0.05, **, *p*< 0.01, ***, *p*< 0.001).


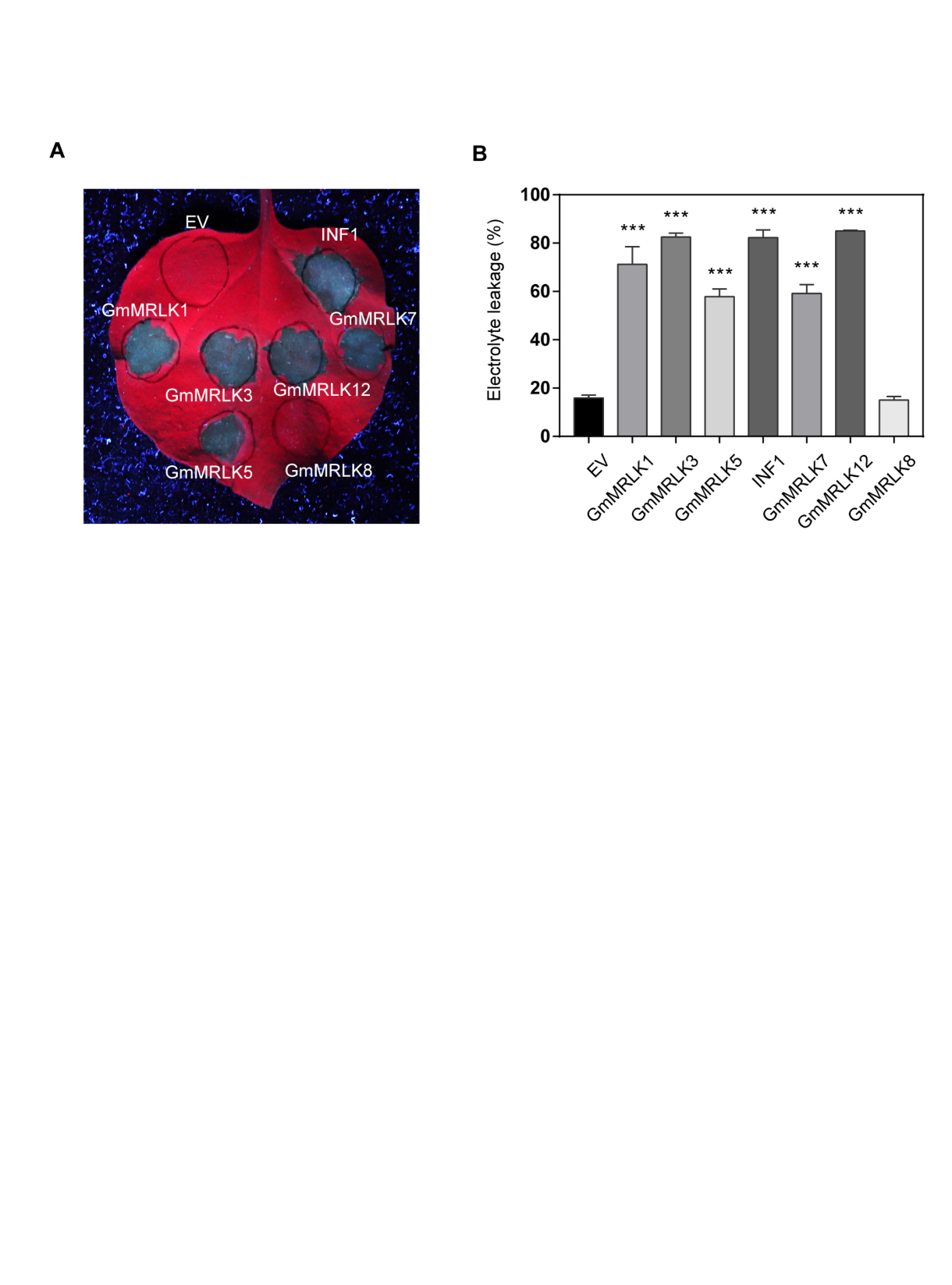


**Figure S6 Expression of GmMRLK1, 3, 5, 7 and 12 in *N. benthamiana* plants caused strong cell death phenotype.**

The indicated GmMRLKs were expressed in *N. benthamiana* by *Agrobacterium*-mediated transient expression for 3-4 days. The cell death phenotype were visualized under UV light (A) and examined by ion leakage assay (B) (Mean±SD, n≥8, Student’s t-test; ***, *p*< 0.001).


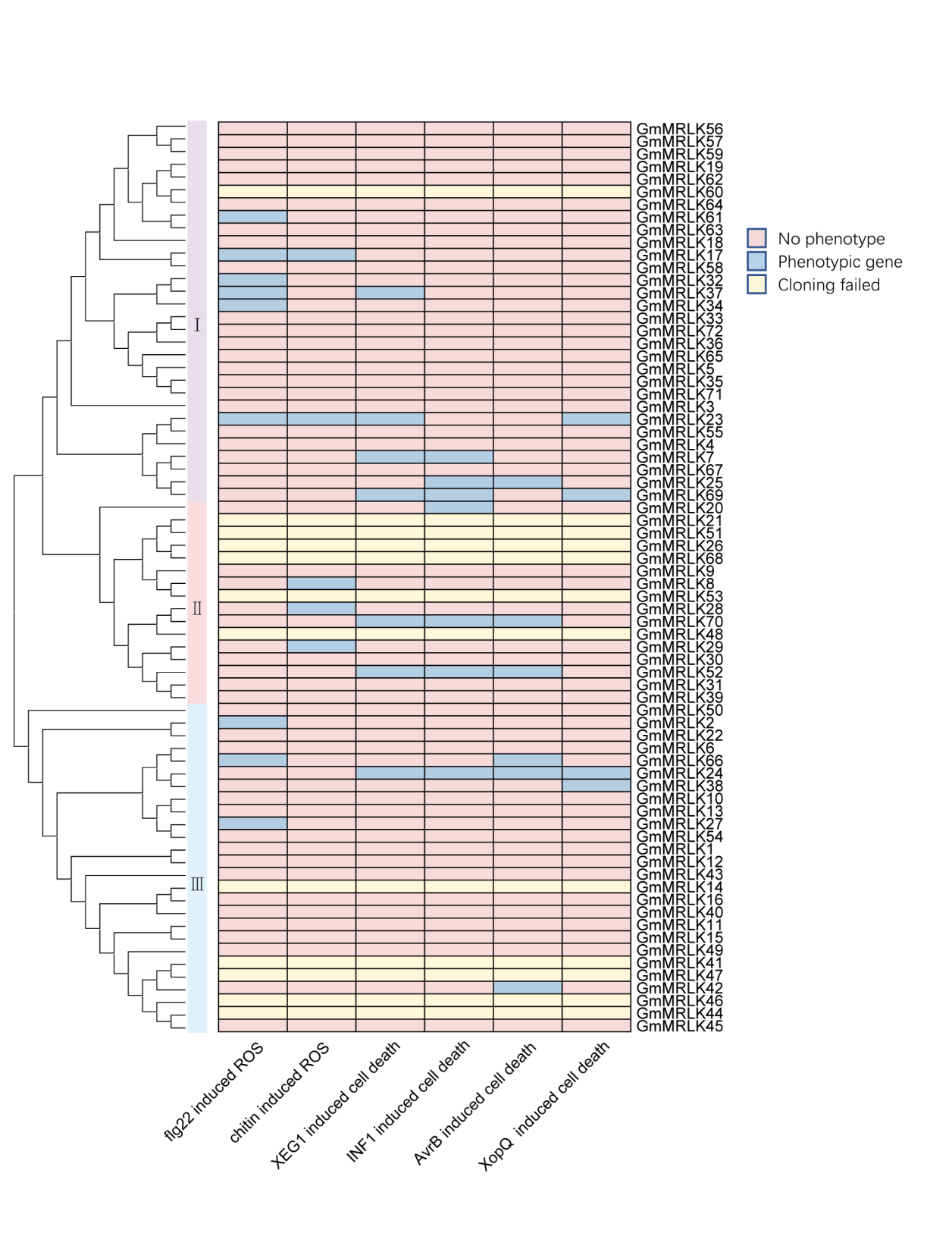


**Figure S7 Summary of different screening systems for *GmMRLK*s genes.**

Results of all the immunity-related assays including *GmMRLK* gene expression in response to *P. sojae* treatment, flg22- and chitin-induce ROS burst, XEG1- and INF-induce cell death, AvrB- and XopQ-induced HR were summarized in the chart. The heatmap was illustrated by TB tools. The blue rectangle means *GmMRLKs* were involved in the indicated immune response. The pink rectangle means no phenotype and yellow rectangle indicated the genes were not cloned.

**

**

**Figure S8 Transient expression of GmMRLK7 and GmMRLK25 in soybean hairy roots greatly promoted plant susceptibility to *P. sojae*.**

The indicated GmMRLKs and EV were transiently expressed in root soybean hairy roots by *A. rhizogenes* -mediated expression for 25 days, infected with zoospores of *P. sojae* for 36 hrs, and the phenotype was visualized by microscope.

**Supplementary Table 2 | Primers used in the study.**

| **Primers** | **sequence** |
| --- | --- |
| GmMRLK1-HA-F | ACGGGGGACGAGCTCGGTACC ATGCCAAGTTCGATCGGGT |
| GmMRLK1-HA-R | AACATCGTATGGGTAGTCGAC TCTTGGAAAAGGAGAGGTG |
| GmMRLK2-HA-F | ACGGGGGACGAGCTCGGTACC ATGATGGAGTTACCAGATA |
| GmMRLK2-HA-R | AACATCGTATGGGTAGTCGAC TCTTGGCTGTGGGTGGGAC |
| GmMRLK3-HA-F | ACGGGGGACGAGCTCGGTACC ATGCGTAATAAAACTAAGC |
| GmMRLK3-HA-R | AACATCGTATGGGTAGTCGAC AGTTGCATTAATTTCTTGT |
| GmMRLK4-HA-F | ACGGGGGACGAGCTCGGTACC ATGCCACTTGGTTTATACA |
| GmMRLK4-HA-R | AACATCGTATGGGTAGTCGAC CCGACCAACAGGCATCATG |
| GmMRLK5-HA-F | ACGGGGGACGAGCTCGGTACC ATGAGCTTCCTCAGTATCA |
| GmMRLK5-HA-R | AACATCGTATGGGTAGTCGAC ATTCCATTTGGTCACCATG |
| GmMRLK6-HA-F | ACGGGGGACGAGCTCGGTACC ATGGAGAGTATCTTACTCT |
| GmMRLK6-HA-R | AACATCGTATGGGTAGTCGAC TCTTGCTGTGGGCTGTGCA |
| GmMRLK7-HA-F | ACGGGGGACGAGCTCGGTACC ATGCAATATTCCAGTGCAA |
| GmMRLK7-HA-R | AACATCGTATGGGTAGTCGAC TCGTCCTTTTGGACTCGCA |
| GmMRLK8-HA-F | ACGGGGGACGAGCTCGGTACC ATGGCGATTCTTCAGCTCC |
| GmMRLK8-HA-R | AACATCGTATGGGTAGTCGAC TCTGGCGTCTTCCATTTTC |
| GmMRLK9-HA-F | ACGGGGGACGAGCTCGGTACC ATGGCGATTGCTATGCTCC |
| GmMRLK9-HA-R | AACATCGTATGGGTAGTCGAC TCTGGCGTCATCAATTTTC |
| GmMRLK10-HA-F | ACGGGGGACGAGCTCGGTACC ATGGATTTATCTTCTCTTT |
| GmMRLK10-HA-R | AACATCGTATGGGTAGTCGAC TCTTGCTGATGGAAGGCAA |
| GmMRLK11-HA-F | ACGGGGGACGAGCTCGGTACC ATGATACATCAAACAATGA |
| GmMRLK11-HA-R | AACATCGTATGGGTAGTCGAC CCTAGCTGGGGGACCAAGT |
| GmMRLK12-HA-F | ACGGGGGACGAGCTCGGTACC ATGGCAAGTTCAATCGGGT |
| GmMRLK12-HA-R | AACATCGTATGGGTAGTCGAC TCTTGGGAAAGGATAGGTG |
| GmMRLK13-HA-F | ACGGGGGACGAGCTCGGTACC ATGGATTTATCTTCTCTCT |
| GmMRLK13-HA-R | AACATCGTATGGGTAGTCGAC TCTTGCCGATGGAAGGCAA |
| GmMRLK15-HA-F | ACGGGGGACGAGCTCGGTACC ATGATGGGAATGTTATTGC |
| GmMRLK15-HA-R | AACATCGTATGGGTAGTCGAC CCTAGCTGGGGGACCAAGT |
| GmMRLK16-HA-F | ACGGGGGACGAGCTCGGTACC ATGGAAAGAGAAACACGAC |
| GmMRLK16-HA-R | AACATCGTATGGGTAGTCGAC CCTCGCTAGAGAACTCTCT |
| GmMRLK17-HA-F | ACGGGGGACGAGCTCGGTACC ATGGATACAAGTGGCACAC |
| GmMRLK17-HA-R | AACATCGTATGGGTAGTCGAC AGATTCCTTACTGCCATAG |
| GmMRLK18-HA-F | ACGGGGGACGAGCTCGGTACC ATGGAAAAAACTTGCACTG |
| GmMRLK18-HA-R | AACATCGTATGGGTAGTCGAC TCGTCCCTTGGGATCATTA |
| GmMRLK19-HA-F | ACGGGGGACGAGCTCGGTACC ATGGATTGGAATACAGGGT |
| GmMRLK19-HA-R | AACATCGTATGGGTAGTCGAC ATCTGAAACAGGTAGAGTA |
| GmMRLK20-HA-F | ACGGGGGACGAGCTCGGTACC ATGGGGAGTGTTCTTGGGG |
| GmMRLK20-HA-R | AACATCGTATGGGTAGTCGAC ATCAGATTCAGCTGTGTGT |
| GmMRLK22-HA-F | ACGGGGGACGAGCTCGGTACC ATGATGGAGTTACCAGATA |
| GmMRLK22-HA-R | AACATCGTATGGGTAGTCGAC TCTTGGCTGTGGATGGGAT |
| GmMRLK23-HA-F | ACGGGGGACGAGCTCGGTACC ATGATGACGAGCATGAATC |
| GmMRLK23-HA-R | AACATCGTATGGGTAGTCGAC ACGACCTTTTGGATTCATG |
| GmMRLK24-HA-F | ACGGGGGACGAGCTCGGTACC ATGCAGAATAGGGTACCCT |
| GmMRLK24-HA-R | AACATCGTATGGGTAGTCGAC TCGTGCAGTAGGCTGTCCA |
| GmMRLK25-HA-F | ACGGGGGACGAGCTCGGTACC ATGTACATCCACGTGTGTC |
| GmMRLK25-HA-R | AACATCGTATGGGTAGTCGAC TCGTCCTTTTGGATTGGCA |
| GmMRLK27-HA-F | ACGGGGGACGAGCTCGGTACC ATGGGTTTATGTTCTCTTT |
| GmMRLK27-HA-R | AACATCGTATGGGTAGTCGAC TCTGGCTGAGGGGAGGCAA |
| GmMRLK28-HA-F | ACGGGGGACGAGCTCGGTACC ATGAGGGATTGCAGAGAAA |
| GmMRLK28-HA-R | AACATCGTATGGGTAGTCGAC CCTCCCTTCAGACTTCACC |
| GmMRLK29-HA-F | ACGGGGGACGAGCTCGGTACC ATGTTGAAGATGGAACTTA |
| GmMRLK29-HA-R | AACATCGTATGGGTAGTCGAC TCTTCCACGAGGATTTACC |
| GmMRLK30-HA-F | ACGGGGGACGAGCTCGGTACC ATGGTGATGATGAAACTTG |
| GmMRLK30-HA-R | AACATCGTATGGGTAGTCGAC CTCCAAATGAATAAACATC |
| GmMRLK31-HA-F | ACGGGGGACGAGCTCGGTACC ATGGGGAAGATAGAAAAAA |
| GmMRLK31-HA-R | AACATCGTATGGGTAGTCGAC CCTGCCATTGAGATTGCTA |
| GmMRLK32-HA-F | ACGGGGGACGAGCTCGGTACC ATGACGTTCCTCAGTATCA |
| GmMRLK32-HA-R | AACATCGTATGGGTAGTCGAC ACGTGGCTTTGGATCCACA |
| GmMRLK33-HA-F | ACGGGGGACGAGCTCGGTACC ATGATATCAACCGTGAGAT |
| GmMRLK33-HA-R | AACATCGTATGGGTAGTCGAC ATTTCCTCTTTTTATTTCA |
| GmMRLK34-HA-F | ACGGGGGACGAGCTCGGTACC ATGACGTTCCTCAGTATCA |
| GmMRLK34-HA-R | AACATCGTATGGGTAGTCGAC GAGGTGGCCGAGCGCACAA |
| GmMRLK35-HA-F | ACGGGGGACGAGCTCGGTACC ATGAGGTTCAACAGTATCA |
| GmMRLK35-HA-R | AACATCGTATGGGTAGTCGAC ATTTCCTCTTTTTACATTC |
| GmMRLK36-HA-F | ACGGGGGACGAGCTCGGTACC ATGAGGCTCCTTAGCATCA |
| GmMRLK36-HA-R | AACATCGTATGGGTAGTCGAC ATTTCCTCTTTTTATTTCA |
| GmMRLK37-HA-F | ACGGGGGACGAGCTCGGTACC ATGAGGCTCCTTAGCATCA |
| GmMRLK37-HA-R | AACATCGTATGGGTAGTCGAC ACGTGGCTTTGGATCCACA |
| GmMRLK38-HA-F | ACGGGGGACGAGCTCGGTACC ATGCTAACGGATTTTATCT |
| GmMRLK38-HA-R | AACATCGTATGGGTAGTCGAC TCGTGCAATAGGCTGTGCA |
| GmMRLK39-HA-F | ACGGGGGACGAGCTCGGTACC ATGGGGAAGATAGAAAAAA |
| GmMRLK39-HA-R | AACATCGTATGGGTAGTCGAC CCTACCATTGAGATTGCTA |
| GmMRLK40-HA-F | ACGGGGGACGAGCTCGGTACC ATGGAAGGACACTTTCTAT |
| GmMRLK40-HA-R | AACATCGTATGGGTAGTCGAC TCTTTGTAGAGAGCTCT |
| GmMRLK42-HA-F | ACGGGGGACGAGCTCGGTACC ATGGCGCTCGTATATCTGT |
| GmMRLK42-HA-R | AACATCGTATGGGTAGTCGAC TCTAGCCAGTGGAGTGAAT |
| GmMRLK43-HA-F | ACGGGGGACGAGCTCGGTACC ATGGATGGAAAATGGAGAT |
| GmMRLK43-HA-R | AACATCGTATGGGTAGTCGAC TCTTGCATCTGGGCCACAC |
| GmMRLK49-HA-F | ACGGGGGACGAGCTCGGTACC ATGAGAAATATGGGAATGT |
| GmMRLK49-HA-R | AACATCGTATGGGTAGTCGAC CCTGGCCTGGGGAACAACT |
| GmMRLK50-HA-F | ACGGGGGACGAGCTCGGTACC ATGACCTCATGGACTTCAG |
| GmMRLK50-HA-R | AACATCGTATGGGTAGTCGAC GTTCTCATTTTGACAAGAT |
| GmMRLK52-HA-F | ACGGGGGACGAGCTCGGTACC ATGGAAGTAGAAAACAAAG |
| GmMRLK52-HA-R | AACATCGTATGGGTAGTCGAC CCTTCCTTGAAAGTTGGCT |
| GmMRLK54-HA-F | ACGGGGGACGAGCTCGGTACC ATGGGTTTATGTTCTCTTT |
| GmMRLK54-HA-R | AACATCGTATGGGTAGTCGAC TCTTGCTGAGGGGAGGCAA |
| GmMRLK55-HA-F | ACGGGGGACGAGCTCGGTACC ATGAGGAGCATGAATCGGT |
| GmMRLK55-HA-R | AACATCGTATGGGTAGTCGAC ACGACCTTTTGGATTCATT |
| GmMRLK56-HA-F | ACGGGGGACGAGCTCGGTACC ATGGACACAACTAGCACCC |
| GmMRLK56-HA-R | AACATCGTATGGGTAGTCGAC TCGTCCCTTTGGATCCTTA |
| GmMRLK57-HA-F | ACGGGGGACGAGCTCGGTACC ATGGGCACAACTTGCGTAC |
| GmMRLK57-HA-R | AACATCGTATGGGTAGTCGAC TCGTCCCTTTGGATCCTTA |
| GmMRLK58-HA-F | ACGGGGGACGAGCTCGGTACC ATGGTACCCCCAGTTATTC |
| GmMRLK58-HA-R | AACATCGTATGGGTAGTCGAC TCGTCCCTTTGGATCCTTA |
| GmMRLK59-HA-F | ACGGGGGACGAGCTCGGTACC ATGGACACAACTGGCACCC |
| GmMRLK59-HA-R | AACATCGTATGGGTAGTCGAC ACCTCTCTCCTCAGAGTCC |
| GmMRLK61-HA-F | ACGGGGGACGAGCTCGGTACC ATGGACACAAATTGCATAC |
| GmMRLK61-HA-R | AACATCGTATGGGTAGTCGAC TCGTCCATTTGGATCCTTA |
| GmMRLK62-HA-F | ACGGGGGACGAGCTCGGTACC ATGGACACTACTTGCACAC |
| GmMRLK62-HA-R | AACATCGTATGGGTAGTCGAC CCGTCGCTTTGGATTCTTA |
| GmMRLK63-HA-F | ACGGGGGACGAGCTCGGTACC ATGTGTGCTGCTTCCATTG |
| GmMRLK63-HA-R | AACATCGTATGGGTAGTCGAC TCGTCCTTTTGGATCCTTA |
| GmMRLK64-HA-F | ACGGGGGACGAGCTCGGTACC ATGGACACAACTGGCACCC |
| GmMRLK65-HA-R | AACATCGTATGGGTAGTCGAC TCGTCCCTTTGGATTCTTA |
| GmMRLK65-HA-F | ACGGGGGACGAGCTCGGTACC ATGACGTTCCTCGGTATCA |
| GmMRLK65-HA-R | AACATCGTATGGGTAGTCGAC CAATTTGTTTTTGCCCATG |
| GmMRLK66-HA-F | ACGGGGGACGAGCTCGGTACC ATGGAGAGTATCTTCCTCT |
| GmMRLK66-HA-R | AACATCGTATGGGTAGTCGAC TCTTGCTGTAGGCTGTGCA |
| GmMRLK67-HA-F | ACGGGGGACGAGCTCGGTACC ATGCAACATTCCAGTGCAA |
| GmMRLK67-HA-R | AACATCGTATGGGTAGTCGAC TCGTCCTTTTGGATTGACA |
| GmMRLK69-HA-F | ACGGGGGACGAGCTCGGTACC ATGCATTCAACCATTGCGT |
| GmMRLK69-HA-R | AACATCGTATGGGTAGTCGAC GTGGCTAAGGTCATGCTCA |
| GmMRLK70-HA-F | ACGGGGGACGAGCTCGGTACC ATGAAGGATTGCAGAGAAA |
| GmMRLK70-HA-R | AACATCGTATGGGTAGTCGAC CCTCCCTTCAGACTTCACC |
| GmMRLK71-HA-F | ACGGGGGACGAGCTCGGTACC ATGAGGTTCAACAGTATCA |
| GmMRLK71-HA-R | AACATCGTATGGGTAGTCGAC ATTTCCTCTTTTTACATTC |
| GmMRLK72-HA-F | ACGGGGGACGAGCTCGGTACC ATGATATCAACCGTGAGAT |
| GmMRLK72-HA-R | AACATCGTATGGGTAGTCGAC ATTTCCTCTTTTTATTTCA |
| qPCR-GmMRLK12-F | TCTTAATGCAGTTGAGATTTTCGT |
| qPCR-GmMRLK12-R | CATGGATCACCCACCCAATTT |
| qPCR-GmMRLK15-F | TTGGCGAAAAACAACCTTACTG |
| qPCR-GmMRLK15-R | CTGATGCTACTACCGGGATAACT |
| qPCR-GmMRLK26-F | GGGCATGGTGGAGCAAATTGTT |
| qPCR-GmMRLK26-R | TCCCTTTGTTGACCACTTTCTT |
| qPCR-GmMRLK48-F | ACTCCAAACTGGCTGCTTCA |
| qPCR-GmMRLK48-R | ACCTCAGAAGGGCCAATACT |
| qPCR-GmMRLK58-F | CTGGAGGACGAACAATCCCT |
| qPCR-GmMRLK58-R | CTGGAGGACGAACAATCCCT |
| qPCR-GmMRLK68-F | ACTCTTCAAGGAACCAAACCTCT |
| qPCR-GmMRLK68-R | AGTGGAATCTCCCACGAAGA |
| qPCR-GmMRLK71-F | GTTCAGGAACTGGGCTGGTC |
| qPCR-GmMRLK71-R | GTGTTTGTGCCCATGACACG |
| qPCR-GmMRLK1-F | GGCTATAGACCCCCATTTAAAGTC |
| qPCR-GmMRLK1-R | CTCAAGCTGTTCCACTTCAGC |
| qPCR-GmMRLK4-F | TGTCGTGATGGTTCCCAAAGT |
| qPCR-GmMRLK4-R | TCTCCACACCATTCGAGAAAGA |
| qPCR-GmMRLK55-F | TCACGTCGTCGCAGGCA |
| qPCR-GmMRLK55-R | AGTTGTTTGTGGCAGCCTTG |
| qPCR-GmMRLK2-F | TCTGCTTAGTGAAGGGGGAG |
| qPCR-GmMRLK2-R | TCACTTGGGTCGGTTGGGAA |
| qPCR-GmMRLK5-F | CGGTAGTTCCAACCCTGTGG |
| qPCR-GmMRLK5-R | GAGAGACGAGATGGCGATGG |
| qPCR-GmMRLK6-F | CAGTTGTAAATGAGACAAGGAAGC |
| qPCR-GmMRLK6-R | AGAAGCTGGCCCTCAATAGG |
| qPCR-GmMRLK27-F | AACCTGTCAAGAAGGAATCTCA |
| qPCR-GmMRLK27-R | ACCCAAGTAAGATGGCAATGGA |
| qPCR-GmMRLK42-F | CATGCGAATCTCACCTCCCT |
| qPCR-GmMRLK42-R | GTACTCCAATCCTAAGGCTGC |
| qPCR-GmMRLK43-F | GTTGACATCATCGGTGGGTA |
| qPCR-GmMRLK43-R | TTGTGCCACCTAATCCACTG |
| qPCR-GmMRLK53-F | AACAGCTCTGCTAGTGCCAG |
| qPCR-GmMRLK53-R | GGCAGGCAACTCCTTAGACA |
| qPCR-GmMRLK3-F | TTGCGGCAATATTCGAACTAAG |
| qPCR-GmMRLK3-R | AGAATGAAAAACGTGAGCAGTC |
| qPCR-GmMRLK11-F | GCTTGTTAAGATCACCGATGAC |
| qPCR-GmMRLK11-R | GAGTGTCGTCAATAATTCCGTG |
| qPCR-GmMRLK40-F | GCTTCATCCAACTATGCTCATG |
| qPCR-GmMRLK40-R | CTACGTCAAATCGTGCCAATAG |
| qPCR-GmMRLK23-F | GCTGATCCAGATGTGAAGTTTG |
| qPCR-GmMRLK23-R | TAATTCGTGTTGATCTCGGGAT |
| pBin-GmMRLK25GFP-F | TTTACGAACGATAG GGTACC ATGTACATCCACGTGTGTCT |
| pBin-GmMRLK25GFP-R | CCCTTGCTCACCATGGATCCTCGTCCTTTTGGATTGGCAA |
| pBin-GmMRLK20GFP-F | TTTACGAACGATAG GGTACC ATGGGGAGTGTTCTTGGGGA |
| pBin-GmMRLK20GFP-R | CCCTTGCTCACCATGGATCCATCAGATTCAGCTGTGTGTT |
| pBin-GmMRLK7GFP-F | TTTACGAACGATAGGGTACCATGCAATATTCCAGTGCAAA |
| pBin-GmMRLK7GFP-R | CCCTTGCTCACCATGGATCCTCGTCCTTTTGGACTCGCAA |
| Glyma.16g12160-Nluc-F | ACGGGGGACGAGCTCGGTACC ATGGCACAACGTTCTCATG |
| Glyma.16g12160-Nluc-R | AACATCGTATGGGTAGTCGAC TTTTTTCCCACCCCACGCAA |
| Glyma.08G083300Nluc-F | AACATCGTATGGGTAGTCGACTAACAAGCCAGCTTCCAAGAGAT |
| Glyma.08G083300Nluc-R | AACATCGTATGGGTAGTCGACACAAGACAGTGTTGTTTGAAGCTTCA |
| Glyma.02g270700Nluc-F | ACGGGGGACGAGCTCGGTACCATGGAACACAGTTTCAGATT |
| Glyma.02g270700Nluc-R | AACATCGTATGGGTAGTCGACTTTACCAGACATTAGATTTG |
| GmMRLK23-Ccluc-F | CGGGGGACGAGCTCGGTACC ATGATGACGAGCATGAATCG |
| GmMRLK23-Ccluc-R | GCGTACGAGATCTGGTCGAC ACGACCTTTTGGATTCATGA |
| GmMRLK2-Ccluc-F | C GGGGGAC GAGCTC GGTACCATGATGGAGTTACCAGATAT |
| GmMRLK2-Ccluc-R | GCGTACGAGATCTGGTCGACTCTTGGCTGTGGGTGGGACA |
| GmMRLK25-Ccluc-F | CGGGGGACGAGCTCGGTACCATGTACATCCACGTGTGTCT |
| GmMRLK25-Ccluc-R | GCGTACGAGATCTGGTCGACTCGTCCTTTTGGATTGGCAA |
| GmMRLK24-Ccluc-F | CGGGGGACGAGCTCGGTACCATGCAGAATAGGGTACCCTT |
| GmMRLK24-Ccluc-R | GCGTACGAGATCTGGTCGACTCGTGCAGTAGGCTGTCCAA |

**Supplementary Table 3 | GmMRLK family members in Williams 82 soybean.**

| **Gene name** | **Gene ID** | **Chromosome** | **Amino acid (aa)** | **Mw (kDa)** | **Cloned** |
| --- | --- | --- | --- | --- | --- |
| GmMRLK1 | Glyma.01G004800 | 1 | 901 | 101 | yes |
| GmMRLK2 | Glyma.01G020100 | 1 | 919 | 103 | yes |
| GmMRLK3 | Glyma.02G121900 | 2 | 820 | 92 | yes |
| GmMRLK4 | Glyma.02G122000 | 2 | 647 | 73 | yes |
| GmMRLK5 | Glyma.02G196000 | 2 | 826 | 93 | yes |
| GmMRLK6 | Glyma.03G177600 | 3 | 932 | 104 | yes |
| GmMRLK7 | Glyma.03G247800 | 3 | 869 | 96 | yes |
| GmMRLK8 | Glyma.05G099900 | 5 | 793 | 88 | yes |
| GmMRLK9 | Glyma.05G100000 | 5 | 838 | 94 | yes |
| GmMRLK10 | Glyma.05G144400 | 5 | 931 | 104 | yes |
| GmMRLK11 | Glyma.07G013700 | 7 | 901 | 100 | yes |
| GmMRLK12 | Glyma.07G127100 | 7 | 901 | 101 | yes |
| GmMRLK13 | Glyma.08G100800 | 8 | 933 | 104 | yes |
| GmMRLK14 | Glyma.08G198800 | 8 | 897 | 101 | no |
| GmMRLK15 | Glyma.08G198900 | 8 | 895 | 100 | yes |
| GmMRLK16 | Glyma.08G199400 | 8 | 897 | 101 | yes |
| GmMRLK17 | Glyma.08G248900 | 8 | 842 | 93 | yes |
| GmMRLK18 | Glyma.08G249200 | 8 | 871 | 97 | yes |
| GmMRLK19 | Glyma.08G249400 | 8 | 790 | 89 | yes |
| GmMRLK20 | Glyma.09G024700 | 9 | 852 | 94 | yes |
| GmMRLK21 | Glyma.09G133000 | 9 | 818 | 91 | no |
| GmMRLK22 | Glyma.09G202300 | 9 | 919 | 103 | yes |
| GmMRLK23 | Glyma.09G273300 | 9 | 896 | 98 | yes |
| GmMRLK24 | Glyma.10G048800 | 10 | 923 | 102 | yes |
| GmMRLK25 | Glyma.10G163200 | 10 | 862 | 96 | yes |
| GmMRLK26 | Glyma.10G231500 | 10 | 826 | 92 | no |
| GmMRLK27 | Glyma.11G246200 | 11 | 930 | 104 | yes |
| GmMRLK28 | Glyma.12G074600 | 12 | 837 | 93 | yes |
| GmMRLK29 | Glyma.12G148200 | 12 | 846 | 93 | yes |
| GmMRLK30 | Glyma.12G220400 | 12 | 689 | 76 | yes |
| GmMRLK31 | Glyma.12G235900 | 12 | 878 | 96 | yes |
| GmMRLK32 | Glyma.13G053600 | 13 | 894 | 99 | yes |
| GmMRLK33 | Glyma.13G053700 | 13 | 819 | 91 | yes |
| GmMRLK34 | Glyma.13G053800 | 13 | 702 | 78 | yes |
| GmMRLK35 | Glyma.13G054200 | 13 | 787 | 89 | yes |
| GmMRLK36 | Glyma.13G054300 | 13 | 844 | 94 | yes |
| GmMRLK37 | Glyma.13G054400 | 13 | 896 | 100 | yes |
| GmMRLK38 | Glyma.13G136700 | 13 | 950 | 106 | yes |
| GmMRLK39 | Glyma.13G201400 | 13 | 869 | 95 | yes |
| GmMRLK40 | Glyma.13G352700 | 13 | 881 | 99 | yes |
| GmMRLK41 | Glyma.13G352800 | 13 | 899 | 100 | no |
| GmMRLK42 | Glyma.13G352900 | 13 | 887 | 99 | yes |
| GmMRLK43 | Glyma.15G021300 | 15 | 905 | 102 | yes |
| GmMRLK44 | Glyma.15G021400 | 15 | 897 | 101 | no |
| GmMRLK45 | Glyma.15G021700 | 15 | 898 | 101 | no |
| GmMRLK46 | Glyma.15G021800 | 15 | 892 | 99 | no |
| GmMRLK47 | Glyma.15G021900 | 15 | 888 | 99 | no |
| GmMRLK48 | Glyma.15G042900 | 15 | 741 | 82 | yes |
| GmMRLK49 | Glyma.15G266400 | 15 | 894 | 100 | yes |
| GmMRLK50 | Glyma.16G092600 | 16 | 939 | 104 | yes |
| GmMRLK51 | Glyma.16G179600 | 16 | 773 | 86 | no |
| GmMRLK52 | Glyma.17G102600 | 17 | 861 | 96 | yes |
| GmMRLK53 | Glyma.17G166200 | 17 | 843 | 94 | no |
| GmMRLK54 | Glyma.18G011000 | 18 | 929 | 104 | yes |
| GmMRLK55 | Glyma.18G215800 | 18 | 894 | 98 | yes |
| GmMRLK56 | Glyma.18G269900 | 18 | 869 | 97 | yes |
| GmMRLK57 | Glyma.18G270100 | 18 | 868 | 97 | yes |
| GmMRLK58 | Glyma.18G270600 | 18 | 1123 | 125 | yes |
| GmMRLK59 | Glyma.18G270700 | 18 | 857 | 96 | yes |
| GmMRLK60 | Glyma.18G270800 | 18 | 909 | 103 | no |
| GmMRLK61 | Glyma.18G270900 | 18 | 875 | 97 | yes |
| GmMRLK62 | Glyma.18G271000 | 18 | 863 | 97 | yes |
| GmMRLK63 | Glyma.18G271100 | 18 | 883 | 98 | yes |
| GmMRLK64 | Glyma.18G271200 | 18 | 849 | 95 | yes |
| GmMRLK65 | Glyma.19G033100 | 19 | 1186 | 134 | yes |
| GmMRLK66 | Glyma.19G178400 | 19 | 931 | 104 | yes |
| GmMRLK67 | Glyma.19G245800 | 19 | 866 | 96 | yes |
| GmMRLK68 | Glyma.20G162300 | 20 | 840 | 93 | no |
| GmMRLK69 | Glyma.20G225800 | 20 | 843 | 94 | yes |
| GmMRLK70 | Glyma.U033500 | mitochondria or chloroplasts | 811 | 90 | yes |
| GmMRLK71 | Glyma.U027000 | mitochondria or chloroplasts | 787 | 89 | yes |
| GmMRLK72 | Glyma.U027100 | mitochondria or chloroplasts | 819 | 92 | yes |
